# Towards Developing Novel Prostate Cancer Recurrence Suppressors: Acute Toxicity of Pseurotin A, an Orally Active PCSK9 Axis-Targeting Small-Molecule in Swiss Albino Mice

**DOI:** 10.3390/molecules28031460

**Published:** 2023-02-02

**Authors:** Oliver C. McGehee, Hassan Y. Ebrahim, Ashkan H. Rad, Khaldoun S. Abdelwahed, Ethar A. Mudhish, Judy A. King, Iman E. Helal, Sharon A. Meyer, Khalid A. El Sayed

**Affiliations:** 1 School of Basic Pharmaceutical and Toxicological Sciences, College of Pharmacy, University of Louisiana at Monroe, 1800 Bienville Drive, Monroe, LA 71201, USA; 2 Department of Integrated Medical Education, Thomas F. Frist, Jr. College of Medicine, Belmont University, 1900 Belmont Boulevard, Nashville, TN 37212, USA

**Keywords:** proprotein convertase subtilisin/kexin type 9 (PCSK9), pseurotin A, acute oral toxicity

## Abstract

The proprotein convertase subtilisin kexin type 9 (PCSK9) emerged as a molecular target of great interest for the management of cardiovascular disorders due to its ability to reduce low density lipoprotein (LDL) cholesterol by binding and targeting at LDLR for lysosomal degradation in cells. Preliminary studies revealed that pseurotin A (PsA), a spiro-heterocyclic γ-lactam alkaloid from several marine and terrestrial *Aspergillus* and *Penicillium* species, has the ability to dually suppress the PCSK9 expression and protein–protein interaction (PPI) with LDLR, resulting in an anti-hypercholesterolemic effect and modulating the oncogenic role of PCSK9 axis in breast and prostate cancers progression and recurrence. Thus, a preliminary assessment of the PsA acute toxicity represents the steppingstone to develop PsA as a novel orally active PCSK9 axis modulating cancer recurrence inhibitor. PsA studies for in vitro toxicity on RWPE-1 and CCD 841 CoN human non-tumorigenic prostate and colon cells, respectively, indicated a cellular death shown at a 10-fold level of its reported anticancer activity. Moreover, a Western blot analysis revealed a significant downregulation of the pro-survival marker Bcl-2, along with the upregulation of the proapoptotic Bax and caspases 3/7, suggesting PsA-mediated induction of cell apoptosis at very high concentrations. The Up-and-Down methodology determined the PsA LD_50_ value of >550 mg/kg in male and female Swiss albino mice. Animals were orally administered single doses of PsA at 10, 250, and 500 mg/kg by oral gavage versus vehicle control. Mice were observed daily for 14 days with special care over the first 24 h after dosing to monitor any abnormalities in their behavioral, neuromuscular, and autonomic responses. After 14 days, the mice were euthanized, and their body and organ weights were recorded and collected. Mice plasma samples were subjected to comprehensive hematological and biochemical analyses. Collected mouse organs were histopathologically examined. No morbidity was detected following the PsA oral dosing. The 500 mg/kg female dosing group showed a 45% decrease in the body weight after 14 days but displayed no other signs of toxicity. The 250 mg/kg female dosing group had significantly increased serum levels of liver transaminases AST and ALT versus vehicle control. Moreover, a modest upregulation of apoptotic markers was observed in liver tissues of both animal sexes at 500 mg/kg dose level. However, a histopathological examination revealed no damage to the liver, kidneys, heart, brain, or lungs. While these findings suggest a possible sex-related toxicity at higher doses, the lack of histopathological injury implies that single oral doses of PsA, up to 50-fold the therapeutic dose, do not cause acute organ toxicity in mice though further studies are warranted.

## 1. Introduction

Recently, the inhibition of proprotein convertase subtilisin kexin 9 (PCSK9) has emerged as a promising therapeutic target for reducing low density lipoprotein (LDL) levels. PCSK9 binds to the extracellular domain of the low-density lipoprotein receptor (LDLR), resulting in the receptor not recycling back to the cell membrane and degrading instead in the lysosome [1]. Currently marketed PCSK9 monoclonal antibodies (mAbs), alirocumab and evolocumab have been proven to considerably lower LDL-C levels and subsequent cardiovascular events. However, FDA-approved humanized PCSK9 mAbs are cost-ineffective and are only administered as subcutaneous injections once or twice a month. Thus, several small-molecule orally active PCSK9 inhibitors are being discovered and developed as feasible alternatives to treat atherosclerosis.

There are concerns about the possible negative consequences of very low serum cholesterol on brain health [2,3,4]. PCSK9 inhibitors have been scrutinized since their public launch over a decade ago for their ability to reduce LDL-C levels far below any statin. However, it has been demonstrated that average LDL-C levels of approximately 120–140 mg/dL could be safely reduced to 30–60 mg/dL [2,3]. One of the main issues raised is the connection between low lipoprotein levels and neurocognitive decline [4]. Dysregulation of brain cholesterol metabolism has been tied to many neurodegenerative malignancies. This led to concerns that PCSK9-targeting entities that lower lipids might negatively affect neuronal function or development. However, cholesterol homeostasis in the brain is thought to be independent of the rest of the body as lipoproteins do not cross the blood brain barrier [4]. Circulating PCSK9 mAbs are unable to cross the blood-brain barrier, and thus, potential therapeutic mAbs that reduce PCSK9 are not expected to directly reduce PCSK9 expression levels in the brain [5]. So far, there is no enough evidence to link PCSK9 mAb inhibitors to serious adverse events [6].

It is estimated that marine species comprise 25% of the total species on the planet [7]. In response to their extremely difficult and harsh environments, marine organisms produce potent secondary metabolites with unique chemical structures and bioactivity. These secondary metabolites derived from the abundance of marine species represent a fundamentally untapped resource for drug discovery [8]. Even though discoveries of potential marine-derived therapeutics have been steadily increasing since the advent of SCUBA diving gear in the 1950′s, the number of marine natural products that are under development or marketed as approved drugs are rather few. This can mainly be attributed to difficulties in collecting source organisms, challenges for large-scale production of marine natural products, high potential toxicity of numerous marine compounds, and the lack of scalable extraction procedures resulting in limited quantities insufficient for high-throughput bioassays and subsequent developments [9].

**Scheme 1:**
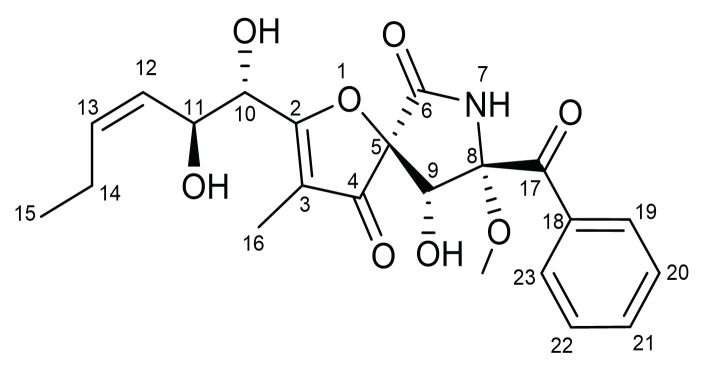
The chemical structure of pseurotin A (PsA).

Pseurotin A (PsA) is a spiroheterocyclic γ-lactam alkaloid (Scheme 1, Supplementary Materials: Figures S1 and S2) first isolated as a fermentation product from *Pseudeurotium ovalis* (strain S2269/F) and later reported in numerous marine and terrestrial *Aspergillus* and *Penicillium* species [10]. PsA competitively inhibited fungal chitin synthase, induced pheochromocytoma PC12 cells differentiation, and was patented as apomorphine antagonist and bacteriostatic food preservative [11]. PsA also showed anti-inflammatory and weak cytotoxic activities. PsA inhibited IgE production and showed anti-seizure activity in animal models. PsA also inhibited osteoclasts and prevented osteoporosis [11]. This novel natural product has been recently reported as a dual inhibitor of PCSK9 secretion and PCSK9 LDLR protein–protein interaction (PPI) [11]. PsA possesses a rare oxygenated 1-oxa-7-azaspiro[4.4]non-2-ene-4,6-dione structural skeleton that is highly substituted with 5 chiral centers [12]. The unique PsA molecular structure mimics a drug-like high selectivity profile at its possible molecular target(s), which was consistent with results of virtual ADMET studies [11,12].

One of the key risk factors for the development of atherosclerosis is elevated LDL levels. The persistent elevation of LDL cholesterol (LDL-C) is a major cause for atherosclerotic cardiovascular disease, and several studies have linked increased serum cholesterol levels with a higher risk of developing cancers [13,14]. However, there are also observations that suggest no association exists between cancer progression and cholesterol [15,16]. This discrepancy reveals that the relationship between cholesterol and cancer might not be a simple two-factor association, and that hypercholesterolemia may only be a contributing factor in some types of cancers but not all. Prostate cancer and breast cancer are two types of cancer with a plethora of clinical analyses and animal experiments that support the relationship between serum cholesterol levels and higher risk of cancer progression and recurrence [17,18,19,20,21]. A meta-analysis highlighted that a non-linear relationship exists between higher dietary cholesterol intake and increased risk of developing breast cancer [22]. Nude mice fed on a high-fat diet (11% fat) developed several-fold higher breast and prostate cancer volumes [11,23]. Studies have revealed PsA’s ability to suppress the progression of hormone dependent BT-474 breast cancer cells in a nude mouse xenograft model [11]. PsA has also been proven in vivo to suppress the progression and recurrence of prostate cancer using a xenograft nude mice model [23].

To the best of our knowledge, the present study is the first to investigate the potential acute toxicity of orally-administered pseurotin A in vivo before it can be validated for efficacy and safety in human clinical trials. Marketed PCSK9 mAb inhibitors evolocumab and alirocumab are currently being used for the treatment of homozygous familial hypercholesterolemia proving the therapeutic potential of targeting PCSK9 [24,25,26]. This study shed light on the safety thresholds and animal exposure limits of PsA, the recently uncovered PCSK9-LDLR axis modulator, which will aid its subsequent validity as a novel orally active small molecule prostate and breast cancer recurrence modulatory entity. This study investigated the in vitro cytotoxicity and acute influence of purified orally-administered PsA treatments on multiple biochemical, hematological, and histopathological parameters in Swiss albino mice.

## 2. Results

### 2.1. In Vitro Cytotoxicity of Pseurotin A

#### 2.1.1. The Effect of PsA on the Viability of Human Non-Tumorigenic Prostate and Colon Cells Using MTT-Based Cell Cytotoxicity

**Figure 1:**
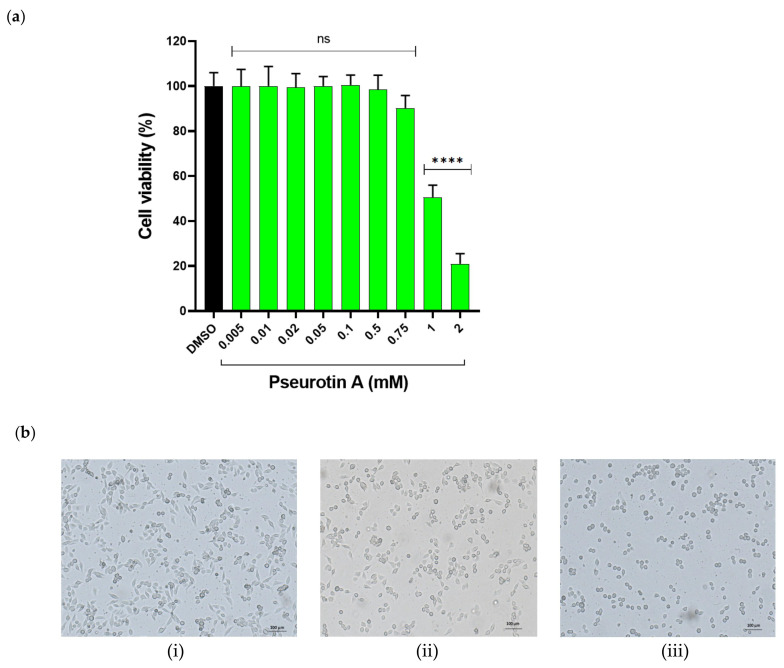
The effect of PsA treatment on the viability of the human immortalized RWPE-1 prostate epithelial cells determined by the standard MTT protocol. (**a**) Bar graph shows dose response of cytotoxic effect of PsA after 24 h treatment. (**b**) Representative images (scale bar embedded) of human immortalized RWPE-1 prostate cells. (**i**) DMSO treated control cells. (**ii**) Cells treated with PsA at 1 mM concentration. (**iii**) Cells treated with PsA at 2 mM level. Data shown as mean ± SD of triplicate wells. **** indicates a statistical significance at *p* < 0.0001 for specified concentrations versus vehicle-treated control cells. ns indicates non-statistical significance at *p* < 0.05.

**Figure 2:**
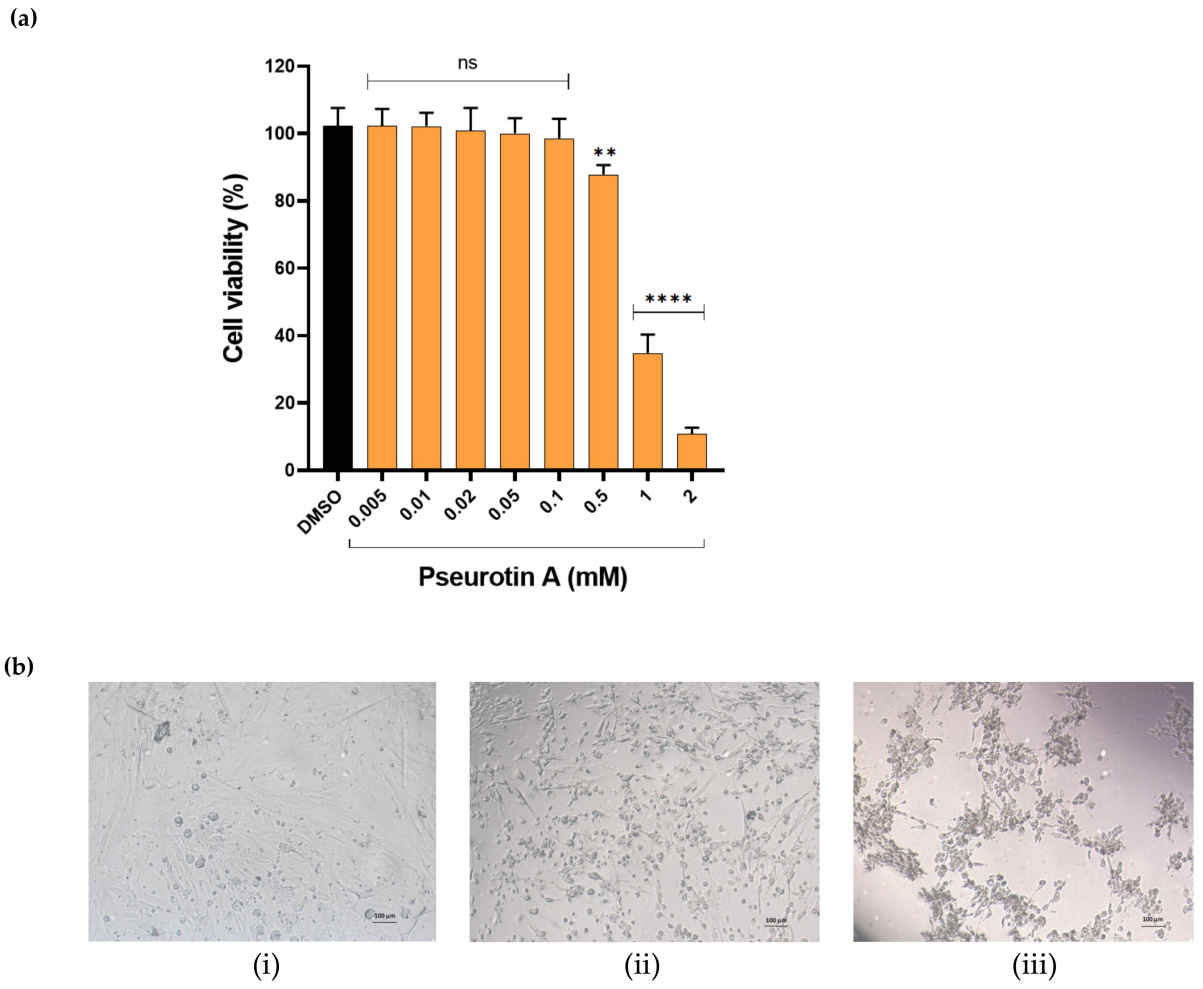
The effect of PsA treatment on the viability of the human CCD 841 CoN non-tumorigenic colon cells determined by the standard MTT protocol. (**a**) Bar graph shows the dose response of cytotoxic effect of PsA over 24 h treatment period. (**b**) Representative images (scale bar embedded) of human CCD 841 CoN non-tumorigenic colon cells over 24 h treatment period. (**i**) Vehicle-treated control cells. (**ii**) Cells treated with PsA at 1 mM concentration. (**iii**) Cells treated with PsA at 2 mM concentration. Data shown as mean ± SD of triplicate wells. ** indicates a statistical significance at *p* < 0.01, while **** indicates a statistical significance at *p* < 0.0001, at specified concentrations vs. DMSO-treated control cells. ns indicates non-statistical significance at *p* < 0.05.

The treatment of human non-tumorigenic prostatic epithelial cells RWPE-1 with different concentrations of PsA induced a dose-dependent cellular death at extremely high concentrations (Figure 1). PsA concentrations ranging from 0.005–0.75 mM did not affect the viability of the treated cells detected by the MTT-based mitochondrial dehydrogenase metabolic activity; meanwhile, 1 and 2 mM concentrations significantly resulted in a 52 and 82% reduction of cell viability, respectively. Moreover, microscopic monitoring of PsA-treated cells revealed a distinct change in cell morphology, including cell rounding and cytoplasmic shrinkage (Figure 1), which is frequently associated with apoptotic cellular death. On the other hand, treatment of human non-tumorigenic colon CCD 841 CoN epithelial cells with different concentrations of PsA showed a similar toxicity pattern to RWPE-1 cells, although colon cells seemed to be more sensitive to PsA treatments (Figure 2). For instance, PsA showed a significant cell toxicity starting at 0.5 mM in CCD 841 CoN cells, while cytotoxicity was observed starting from 0.75 mM in RWPE-1 cells. Moreover, 1 and 2 mM of PsA reduced the cell viability by 65 and 90%, respectively (Figure 2), compared to a 52 and 82% viability in RWPE-1 cells at the same concentrations (Figure 1). Furthermore, the reduction in cell viability was accompanied by cell rounding and shrinking of the cytoplasm, which suggests the likelihood of apoptotic cells death.

#### 2.1.2. The Effect of PsA on Mitochondrial Functionality in Human Normal Prostatic RWPE-1 Cells

**Figure 3:**
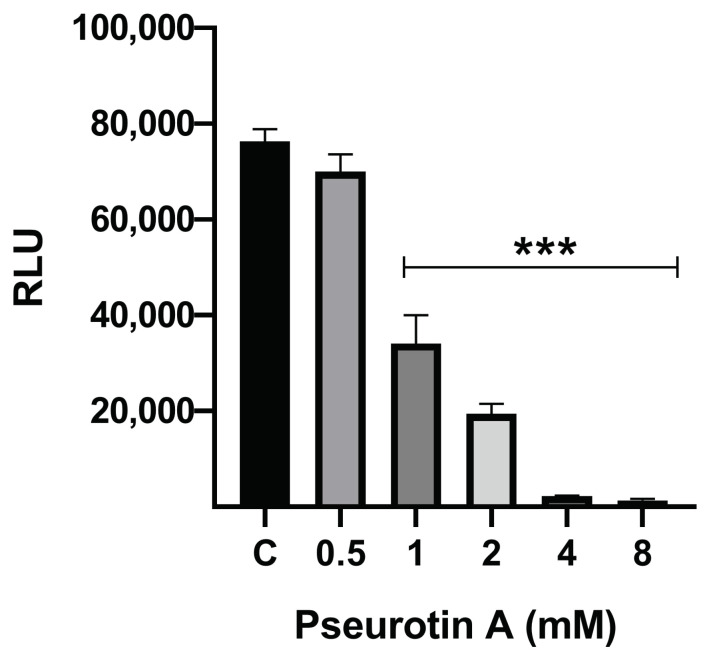
Effect of PsA treatments on the intracellular ATP levels of RWPE-1 cells. Bar graph shows the dose response of mitochondrial functionality of PsA after 24 h treatment. Data shown as mean ± SEM of triplicate wells. *** indicates a statistical significance at *p* < 0.001.

To test for the effect of PsA on mitochondrial functionality, this assay, after inducing cell lysis, used a luciferase tag that binds to ATP in the presence of Mg^2+^ to generate a luminescent signal (RLU) that is proportional to the amount of intracellular ATP present in the cell. Treatment of the RWPE-1 normal human cells with PsA at 8, 4, 2, and 1 mM resulted in significantly reduced ATP upon cell lysis. The 8 mM and 4 mM concentrations were toxic to the cells with a % intracellular ATP of 1.75% and 3.03%, compared to the vehicle control (Figure 3). The 2 mM, 1 mM, and 0.5 mM treatments displayed good dose response with 25.53%, 44.71%, and 91.84% cell viability, respectively, versus vehicle control (Figure 1).

#### 2.1.3. The Effect of PsA on the Apoptotic Signaling Pathway in Human Normal Prostatic RWPE-1 Cells

**Figure 4:**
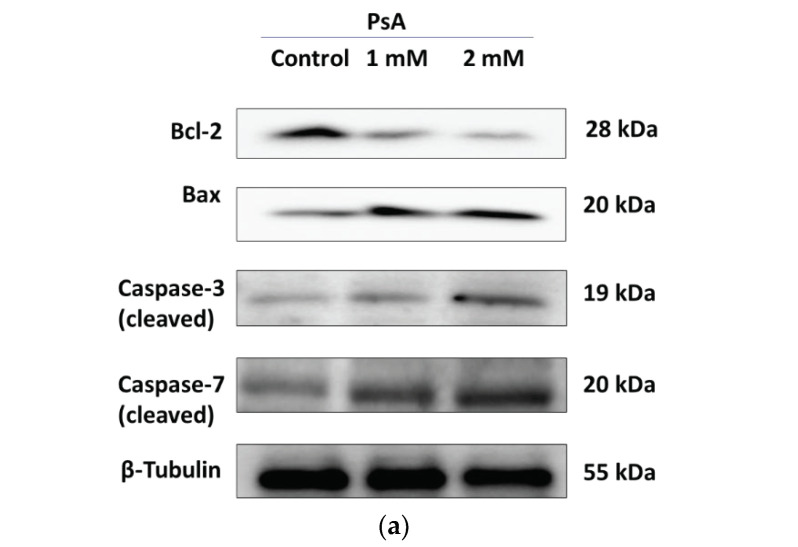

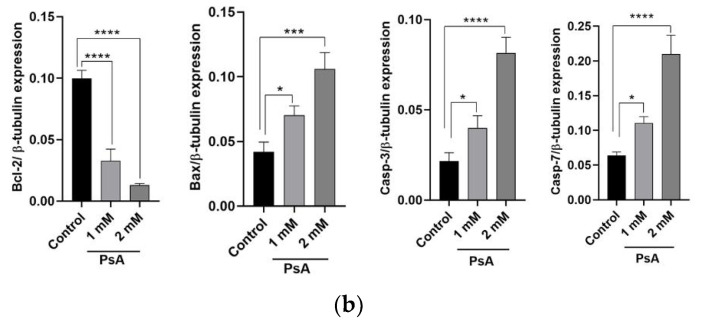
The effect of PsA treatment on apoptotic markers in the human RWPE-1 non-tumorigenic prostate epithelial cells. (**a**) Immunoblots of Bcl-2, Bax, caspase-3 and caspase-7, and β-tubulin from the cell lysate in control, and PsA-treated cells at 1 and 2 mM. (**b**) Densitometric analysis of apoptotic markers protein bands relative to tubulin in each band for B-cell lymphoma 2 (Bcl-2), Bcl-2 associated X-protein (Bax), caspase-3, and caspase-7. * indicates a statistical significance at *p* < 0.05, *** indicates a statistical significance at *p* < 0.001, and **** indicates a statistical significance at *p* < 0.0001 at specified concentrations vs. control.

Treatment of the RWPE-1 normal human cells with PsA at 1 and 2 mM for 24 h induced a significant downregulation of the antiapoptotic Bcl-2 protein (Figure 4). For instance, 1 mM PsA decreased the expression level of Bcl-2 by more than two-fold, while 2 mM reduced Bcl-2 expression by five-fold when compared to untreated control cells. Furthermore, PsA treatments induced the expression levels of the pro-apoptotic Bax in a dose-dependent manner (Figure 4). For example, 1 mM PsA upregulated the Bax expression level by 42%, while 2 mM induced Bax expression by more than 100% (Figure 4) in comparison to Bax level in the untreated control cells. Moreover, proteolytic caspases 3 and 7 were upregulated upon treatment with PsA in a concentration-dependent manner (Figure 4). The expression level of cleaved caspase-3 was increased by 1.5-fold and 4-fold in response to treatments by 1 and 2 mM PsA, respectively (Figure 4). Meanwhile, the expression level of cleaved caspase-7 was doubled in response to PsA treatment at 1 mM and quadrupled at 2 mM concentration (Figure 4).

### 2.2. Determination of LD_50_ of Pseurotin A

The LD_50_ of PsA orally administered was determined to be > 550 mg/kg. A total of four Swiss albino mice (two male and two female) were utilized in this experiment. The first mouse was administered an oral dose of PsA at 175 mg/kg in accordance with the Organization of Economic Co-operation and Development (OECD) guideline 425 [27] suggestion for the starting dose. No behavior, neuromuscular, nor autonomic changes observed at any time points. No animal morbidity was observed over 48 h. Each of the other three mice administered PsA 550 mg/kg oral dose. No morbidity over 48 h nor any changes in behavior, neuromuscular, autonomic responses observed at any time point. Limits of PsA solubility prevented increasing the dose to 2000 mg/kg, which is the upper limit of the Up-and-Down procedure [27].

### 2.3. 14-Day Acute Toxicity Assay of Pseurotin A

#### 2.3.1. PsA Effects on Mouse Body Weight

**Figure 5:**
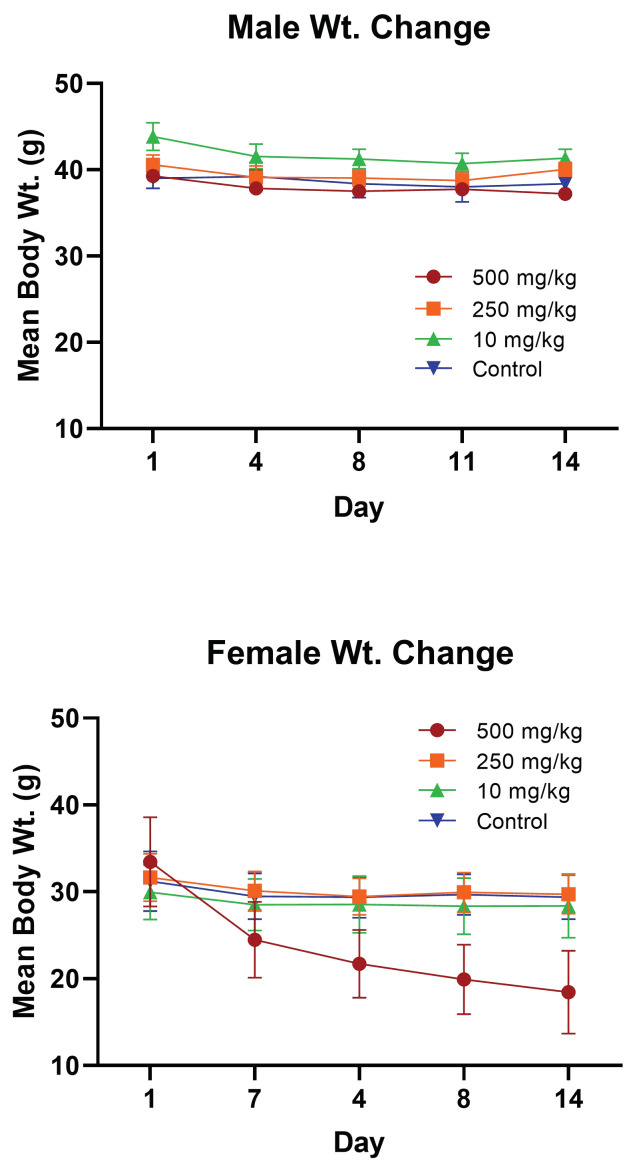
Single oral dose PsA treatment effects on the body weight of male and female Swiss albino mice. Weight was recorded at 1, 4, 8, 11, and 14 d for treatment and control groups. Data shown as mean ± SEM for n = 5/sex/group.

Following a single oral administration of PsA, the bodyweight of the treatment groups for the male mice did not show any significant changes over the five time points measured. The 10 mg/kg and 250 mg/kg PsA treatment groups lost weight over 11 days but a reversal of that trend was observed and the mice gained weight from day 11 to 14. The 500 mg/kg group was the only group that lost notable weight over the entire 14 days (Figure 5). The 500 mg/kg treatment group for the female mice showed a drastic decrease in body weight in the first four days, which continued to decline at each time point measured until the day of sacrifice (Figure 5). The female mice body weight of the 10 mg/kg and 250 mg/kg treatment groups remained constant and did not differ from the control group. This extreme loss in body weight from the female group could be attributed to the PsA’s PCSK9 inhibitory effect that has been shown to lower the amount of LDL cholesterol by a significant margin [11,23].

#### 2.3.2. The Effects of PsA Treatments on Swiss Albino Mice Behavior

The oral administration of PsA for all dosing groups did not negatively affect mice behavior responses (grooming, vocalization, alertness, anti-social), neuromuscular responses (startle response, tonic/clonic convulsions, body posture, gait, righting reflex), nor autonomic responses (diarrhea, cyanosis, piloerection, respiratory rate) at any of the time points observed past the second hour. During the first hour, a few of the mice in the male 500 mg/kg group showed signs of grimace that were measured using the mouse grimace scale [28]. However, after the first 2 h, all mice showed no abnormal behavior nor signs of pain over the remainder of the acute toxicity study.

#### 2.3.3. Evaluation of Relative Animal Organ Weight

Fourteen days after the oral administration of PsA, the mice were sacrificed and their vital organs such as the liver, kidneys, spleen, lungs, heart, and brain were collected and weighed. The kidneys and lungs were both weighed together. The results showed no significant change in weight for treatment groups compared to the control group (Table 1 and Table 2, Dunnett’s test, *p* > 0.05).

#### 2.3.4. Analysis of Mice Hematological Biochemical Parameters after PsA Single Oral Dose

**Figure 6:**
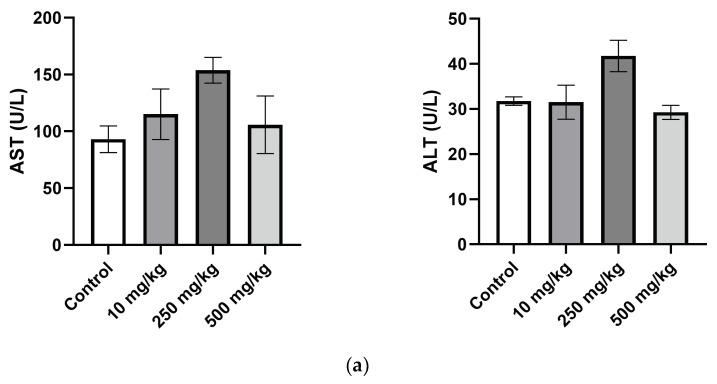

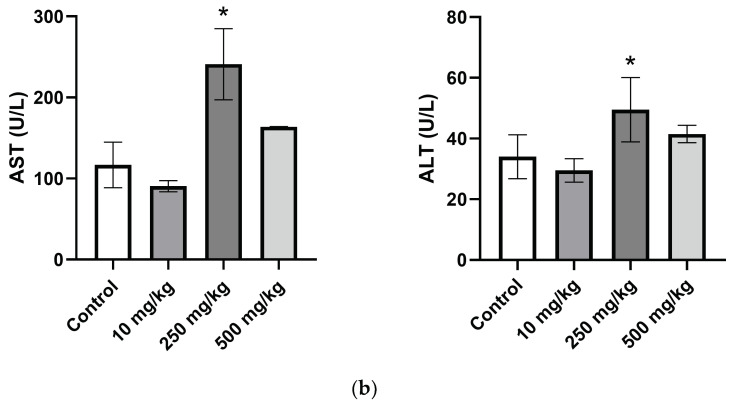
Comparison of the plasma liver enzymes levels in PsA versus vehicle control-treated Swiss albino mice. (**a**) AST and ALT in plasma of male mice. (**b**) AST and ALT in plasma of female mice. Data shown as mean ± SEM for n = 5/sex/group. * indicates a statistical significance at *p* < 0.05.

**Figure 7:**
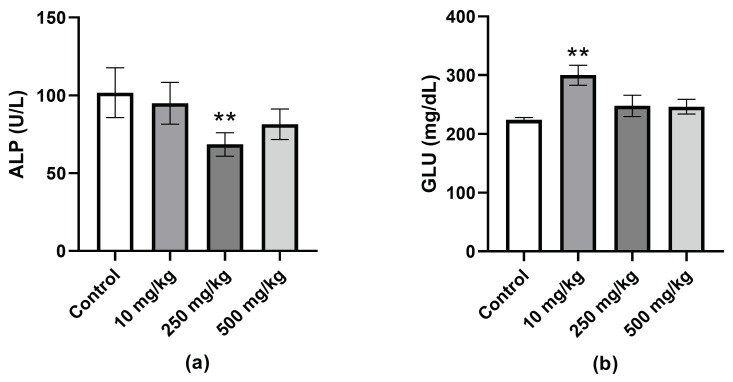
Comparison of the plasma ALP and glucose levels in PsA versus vehicle control-treated Swiss albino mice. (**a**) Plasma ALP levels of female Swiss albino mice treated with PsA. (**b**) Plasma glucose levels of male Swiss albino mice treated with PsA. Data shown as mean ± SEM for n = 5/sex/group. ** indicates a statistical significance at *p* < 0.01.

The results demonstrated that the hematological biochemical parameters aspartate aminotransferase (AST) and alanine aminotransferase (ALT) of the female mice 250 mg/kg dosing group were significantly higher than the parameters of the control mice (Figure 6). The same trend of increased ALT and AST and decreased in ALP was observed in the male 250 mg/kg group (Figure 6) but was not statistically significant (*p* > 0.05). The ALP of the female 250 mg/kg dosed group was significantly lower when compared with the control group. The glucose levels of the 10 mg/kg male group were significantly elevated as compared with vehicle control (Figure 7). None of the other treatment groups, male or female, saw significant changes in glucose levels as compared with that of the control (*p* > 0.05). In addition, these findings showed no changes in renal toxicity markers blood urea nitrogen (BUN) and creatinine from PsA. The creatinine levels remained ≤0.2 mg/dL for all groups and the BUN of the treatment groups did not significantly differ compared to the control (*p* > 0.05) as displayed in Table 3 and Table 4.

#### 2.3.5. Analysis of Hematological Parameters after Single Oral Dose PsA Treatment

**Figure 8:**
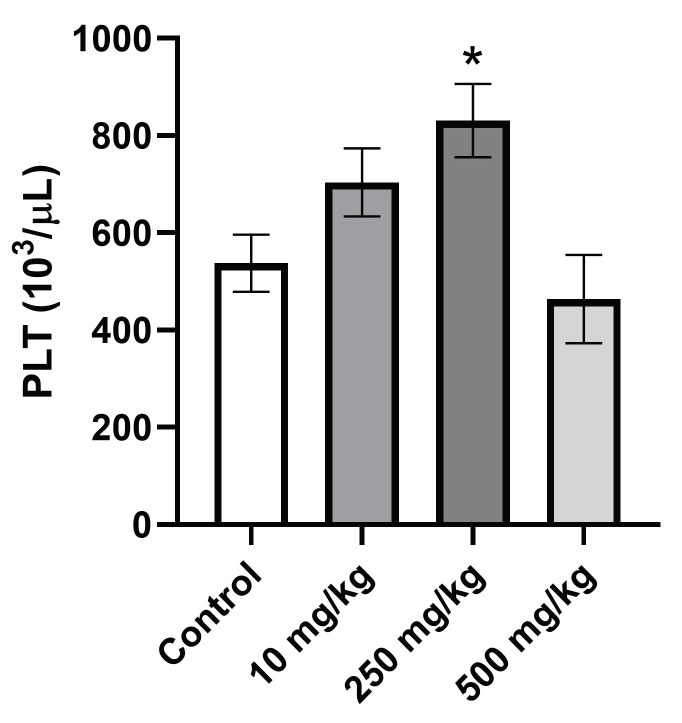
Effects of PsA treatments on male Swiss albino mice platelet count (PLT). Data shown as mean ± SEM for n = 5/group. * indicates a statistical significance at *p* < 0.05.

The effects of up to 500 mg/kg PsA on the hematological parameters of mice following single oral administration are illustrated in Table 5 and Table 6. The red blood cell count (RBC), hemoglobin count (HGB), hematocrit (HCT), mean corpuscular volume (MCV), and mean corpuscular hemoglobin concentration (MCHC) did not show any significant difference versus vehicle control treatment (*p* > 0.05). The white blood cell count (WBC) was lower but non-statistically significant in the 500 mg/kg treatment group compared to control in both mouse genders (*p* > 0.05). The only significant finding amongst the hematological parameters was the increased platelet count (PLT) in the 250 mg/kg male treatment group as compared to vehicle control (Figure 8).

#### 2.3.6. Single Oral Dose PsA Effects on Swiss Albino Mouse Organs Histopathology

**Figure 9:**
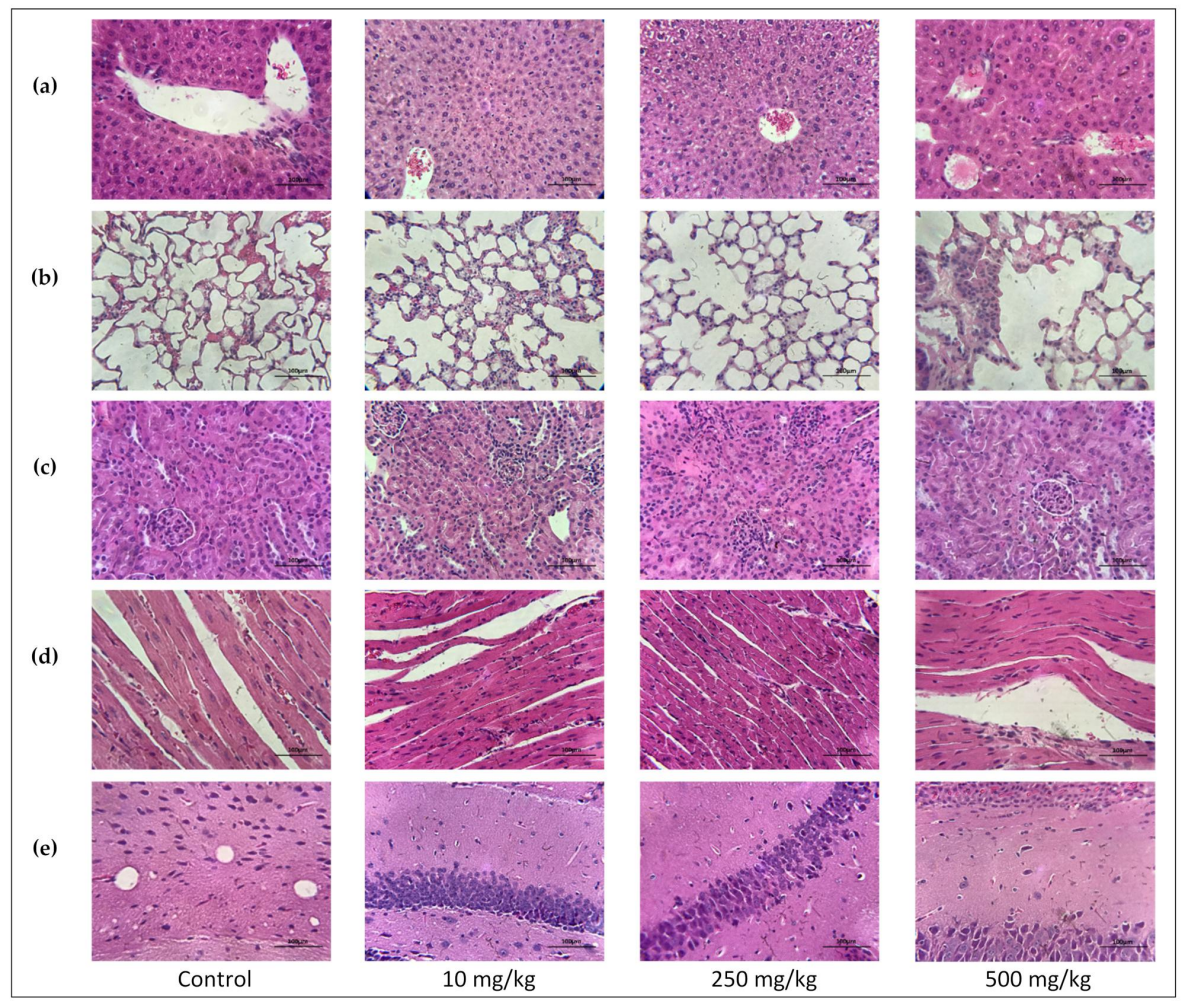
Histopathological examination of Swiss albino female mice organs treated with PsA single oral doses. (**a**) H&E-stained liver sections. (**b**) H&E-stained bronchiolar sections. (**c**) H&E-stained renal cortex sections. (**d**) H&E-stained cardiac muscle sections. (**e**) H&E-stained hippocampal sections. All section images taken at 400× magnification.

After 14 days, PsA and vehicle control-treated animals’ liver, lung, kidney, heart, and brain were subjected to a histopathological examination. Animal organs were analyzed for any signs of gross toxicity including signs of necrosis, apoptosis, tissue lesions, and other organ-specific abnormalities. The results of all five organs up to the highest dose of 500 mg/kg showed no pathological nor morphological changes compared to the vehicle control-treated mice (Figure 9). Greater emphasis was placed on observing the liver and heart. The reasoning behind this is that the liver is the primary xenobiotics detoxifying organ with the highest PCSK9 expression level [29]. It has also been reported that PCSK9 deficiency can potentially impact cardiac lipid metabolism, contributing to the plausible development of heart failure [30]. The results of the histopathological examination with no obvious abnormalities were inconsistent with those of the liver biochemical parameters, specifically, high AST and ALT levels especially in the female 250 mg/kg dosing group. Higher levels of liver transaminases are indicative of potential organ toxicity. This may suggest that the single PsA high dose was not enough to cause histopathological insults.

The structural features of the mouse hepatocytes consisted of large round nuclei (occasionally binucleated), were prominent in the tissue sections, and remained physically intact. The portal veins, bile ducts, and hepatic arteries also remained structurally uncompromised with no signs of sloughed off tissue from neighboring cells found inside (Figure 9). Healthy Kupffer cells were found lining the sinusoids but were not overexpressed in the treatment groups compared to the control group. Coagulation necrosis, characterized by pyknosis in the eosinophilic cytoplasm, was not present in any of the treatment groups. Early signals of inflammation associated with hepatocyte necrosis; cell infiltration, microgranulomas, and granulomas were not present in any of the mice liver sections [31].

The lung tissues were examined for any altercation in the trachea, bronchioles, and alveoli. Special attention was given to the respiratory bronchiole whose lumen presents a thick wall with small pockets of alveoli. The entire alveolus is lined by type I pneumocytes (which perform the gas exchange and make up the majority of alveolar cells), sprinkled with type II pneumocytes (provide surfactant), Clara cells, and resident macrophages (known as dust cells). Any damage to the cells of the alveoli is of particular concern as it is in these alveoli that gas exchange occurs [32]. For the control and each treatment group, the epithelium of the alveolar ducts and alveolar sacs were morphologically intact, and both were free of cellular debris or red blood cell infiltration. The trachea and esophagus were also structurally intact with normal cartilaginous C-rings present in the lumen of the trachea. No injury nor morphological changes observed in the columnar epithelium of the trachea, the lamina propria, seromucous glands, nor perichondrium neither in PsA-treated nor vehicle control groups (Figure 9b).

The kidney tissues were examined for any pathological alteration of the glomerulus, proximal and distal convoluted tubule cells, collecting ducts, and loops of Henle. Healthy tubules were intact with standard cellular shapes. Necrotic tubules will present a compromised monolayer, reduced cell number, and degradation of cell morphology. Injured tubules will lose their circular structure, contain less nuclei, and a thinner monolayer [33]. The glomerulus was structurally homogeneous with an uncompromised Bowman’s capsule. The surrounding proximal and distal tubular cells were assorted circularly with large round nuclei in the correct amount without any morphological changes. The brush border of the proximal convoluted tubule, microvilli on the luminal surface of the epithelial cells, was clearly visible in each treatment group. The collecting ducts were structurally intact and free of sloughed tissue or abundance of red blood cells. There was no observable desquamation, injury, or necrosis of the tubular epithelial cells of either the distal or proximal convoluted tubules. Nor were there signs of regenerative epithelial cells (Figure 9c). These results are in alignment with the biochemical parameters (blood urea and creatinine) results that showed no difference between the PsA treatment group versus vehicle control.

Examined mice hearts showed no morphological changes in the cardiac muscle of the atrium and ventricles. The large oval nuclei of the muscle cells were morphologically normal and centrally located. The intercalated discs, intercellular junctions that connect adjacent cardiac muscle cells, were clearly outlined (Figure 9d). Cardiac muscle demonstrates polygon shaped areas of cardiac muscle fibers (CMF) with easily noticed blood vessels in large intercellular spaces [33]. The CMF were firmly attached with no evidence of muscle fiber derangement, focal lesions, or confluent lesions. No subendocardial necrosis, ventricular wall necrosis, intramyofiber edema, or extensive infiltration of leukocytes were present in the control nor treatment groups [34].

The specific CNS targets of PsA are currently unknown. Thus, we evaluated PsA effects on the commonly CNS-targeted sites including cerebral cortex, cerebellum, and hippocampus [35]. The hippocampus section was given special attention as it is a primary site for neurotoxicity and looking at a hemi-coronal section of the hippocampus through the widest part of the brain can help ensure common CNS target sites will not be overlooked [35]. The H&E stain of the mouse hippocampus demonstrated an evenly arranged and size uniform pyramidal neuronal layer (Figure 9e). Almost all neurons presented a rounded central nucleus with a prominent nucleolus. Healthy glial cells were observed in the molecular layer.

#### 2.3.7. The Effect of PsA on the Apoptotic Signaling Pathway in Liver Tissues of Swiss Albino Mice

**Figure 10:**
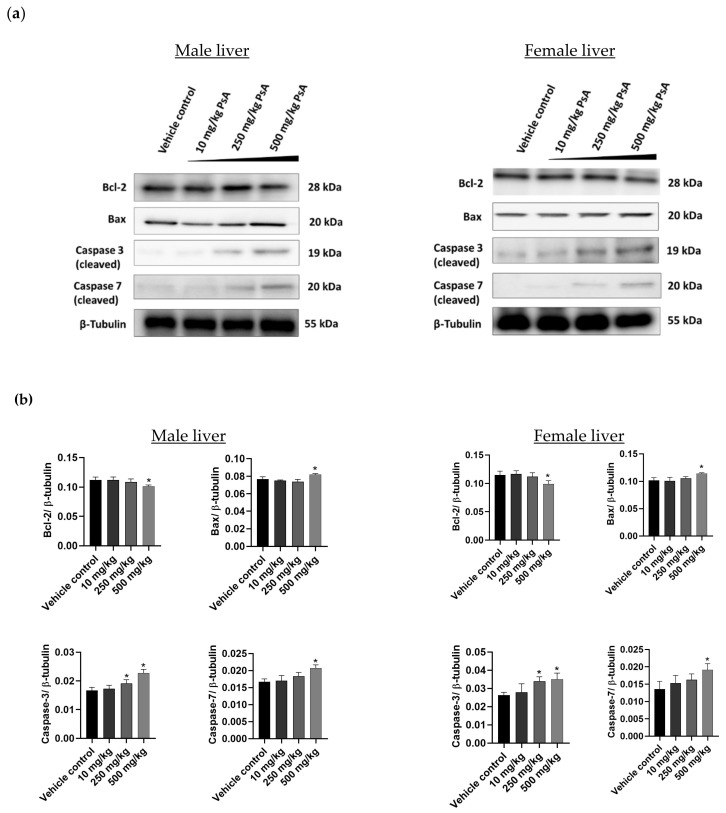
The effect of PsA treatments on the apoptotic markers in Swiss albino mice liver tissue lysates. (**a**) Immunoblots of Bcl-2, Bax, caspase-3, and caspase-7 protein expression 14 days post oral treatments of PsA and vehicle control in male and female mice. (**b**) Densitometric analysis of proteins expression normalized to β-tubulin 14 days post oral treatment of PsA and vehicle control in male and female mice. Data shown as mean ± SD of triplicate wells. * indicates a statistical significance at *p* < 0.05.

The liver is the organ with the highest level of PCSK9 expression in the body [29], qualifying it as a primary target for assessing PsA toxicity. Western blot analyses of hepatic tissue lysates from different treatment groups in both animal sexes revealed the downregulation of the antiapoptotic Bcl-2, concomitantly with upregulation of the apoptotic regulator Bax (Figure 10). In male liver tissues, the expression level of Bcl-2 remained unaffected at 10 and 250 mg/kg single oral doses of PsA versus the vehicle-treated mice (Figure 10). Meanwhile, the Bcl-2 expression level was statistically significantly (*p* < 0.05) lower in liver lysates from mice treated with 500 mg/kg PsA in comparison to lysates from vehicle control (Figure 10). Furthermore, the densitometric analysis of protein blots revealed an approximately 6.2% decrease in Bcl-2 expression level in liver lysates from male mice treated with 500 mg/kg (Figure 10). On the other side, Bax level remained unchanged at doses 10 and 250 mg/kg, while it was upregulated at 500 mg/kg by 9% with respect to vehicle control. Similar patterns observed in liver lysates from female mice, where the reduction in the expression level of Bcl-2 was also statistically significant (*p* < 0.05) at 500 mg/kg and reduction was 7.1% when compared to the expression level in the vehicle-treated control group (Figure 10). Likewise, Bax remained unchanged in liver lysates at doses 10 and 250 mg/kg, and then showed a significant upregulation at 500 mg/kg (Figure 10). The expression levels of executioner apoptotic caspases 3 and 7 remained unaffected at 10 mg/kg dose level, while the 250 and 500 mg/kg treatments induced upregulation of the caspase-3 in both animal genders (Figure 10). Furthermore, the expression level of caspase-7 was significantly overexpressed at 500 mg/kg PsA, while it remained unaffected at 250 mg/kg PsA in both animal genders (Figure 10).

## 3. Discussion

Lipid-lowering therapies, such as statins, continue to be at the forefront of treatment and prevention of cardiovascular events; however, patient’s resistance to continued therapy as well as statin-associated muscle symptoms (SAMS) [36] have spurred the discovery of novel molecular targets and molecules. PCSK9 inhibitors traditionally used to control hypercholesterolemia by reducing LDL-C. PCSK9 proved playing oncogenic driving role in gastric, lung, colon, breast, liver, ovarian, and skin cancers, validating it as a novel molecular target in cancer [11,23]. The marine and terrestrial fermentation product PsA was recently reported as the first small molecule dual inhibitor of PCSK9 expression and PPI [11,23]. Though expectations are high, there are valid concerns over possible adverse side effects of new PCSK9 inhibitors, especially on liver, the main PCSK9 biosynthesizing machinery in the human body. This study, to the best of our knowledge, is the first to analyze and report the acute safety profile in vivo of PsA, the novel PCSK9-LDLR axis modulator. The objectives of this study were to gauge potential toxic effects of PsA through in vitro testing as well as single oral administration in Swiss albino mice; therefore, it preliminarily assessed its prospective safety profile for subsequent developmental stages.

Biosafety appraisal of pharmacologically-validated hits and leads is a critical endpoint at the early stages of the drug development pipeline. In vitro cytotoxicity assays are routinely deployed in cell cultures by assessing cellular death response to tested molecules. Current cellular assays mostly rely on assessing the cell viability as a function of either membrane integrity, mitochondrial functionality, oxidative stress, or cell death [37]. Herein, we adopted three of these approaches to assess the cytotoxic effects of PsA on two non-tumorigenic human epithelial cell lines: the RWPE-1 prostatic and the CCD 841 CoN colon cells. We have previously reported the recurrence suppressing effects of PsA on prostate cancer in animal models [23]. Therefore, the immortalized RWPE-1 human prostatic cells were chosen to mimic the target human prostatic gland. Moreover, the CCD 841 CoN human colon cells also chosen to mimic the GIT cells, since PsA was the first oral small molecule modulator of the PCSK9-LDLR axis [11,23]. Previous in vitro results on prostate cancer cells showed relatively low cytotoxicity, 100–150 µM, but was impressive in suppressing breast and prostate cancers motility and recurrences [11,23]. Thus, we chose doses at effective and relatively higher concentrations to determine the selective effects of PsA (cancer versus immortalized human cells) and to monitor its cytotoxicity at higher concentrations for subsequent preclinical studies. PsA showed no significant effects on the viability of RWPE-1 cells from the concentration range of 0.005 to 0.75 mM (Figure 1), which indicated that PsA lacks cytotoxicity to normal prostate cells at reported anticancer concentrations. Meanwhile, high concentrations at 1 and 2 mM induced significant cell death, indicated by the reduction of cell viability to 49% and 19%, respectively. Moreover, RWPE-1 cells morphological changes were detected at 1 and 2 mM PsA, including cell rounding and cytoplasmic shrinkage (Figure 2), which suggests apoptosis as the main mechanism of cellular death. Furthermore, the viability of CCD 841 CoN colon cells was not much affected when treated with PsA up to 100 µM. PsA 500 µM treatment showed a slight decrease in cell viability (12.3%). Additionally, 1 and 2 mM of PsA induced a significant toxic effect indicated by 38 and 9% cell viability, respectively, at these treatment concentrations. Taken together, this data highlights the broad safety profile of PsA at the cellular level in different human non-tumorigenic cell lines.

To access for changes in mitochondrial functionality induced by PsA, a bioluminescence assay that measures intracellular ATP was utilized. Adenosine triphosphate is the main source of energy for all life on the planet. In cells, the hydrolysis of ATP to ADP releases energy that gets transferred to other molecules. Cellular response to stress can drastically change intracellular ATP, and ATP biosynthesis will be completely halted following cell death. This makes the measuring of intracellular ATP a primary indicator of cell viability. There are multiple validated methods to measure ATP concentration; however, bioluminescence ATP assays are seen as the most rapid, sensitive, and selective [38]. Intracellular ATP levels were significantly downregulated in PsA-treated RWPE-1 cells compared to control cells (Figure 3). This was especially evident in the 4 mM and 8 mM concentrations with a percent intracellular ATP concentration of only 1.75% and 3.03%, respectively, compared to the control. Cells treated with 1 mM PsA showed more pronounced levels of intracellular ATP (44.71%) versus the control, while the 0.5 mM PsA treatment measured ATP 91.84% of the control cells (Figure 3). This data highlights the relative safety of PsA to mitochondrial integrity and function at concentrations below 1 mM.

To determine if apoptotic death is induced by PsA, molecular signaling pathways regulating apoptosis were investigated by a Western blot analysis. It is well documented that the Bcl-2 family of prosurvival proteins regulates apoptosis by controlling the release of cytochrome-c from the inner mitochondrial membrane, which will activate several adaptor proteins such as APAF-1 to form apoptosomes. Bcl-2 was downregulated in PsA-treated cells in comparison to its expression level in control cells indicating an active process of apoptosis at indicated concentrations (Figure 4a). In the same sense, the Bax is recognized for its pro-apoptotic function via opening the voltage-dependent anion channel (VDAC), and thus increasing the permeability of the mitochondrial inner membrane to release cytochrome-c. Immunoblot results of lysates pooled from treated cells revealed the significant upregulation of Bax protein at 1 and 2 mM of PsA when compared to the control cells, proving a dynamic apoptotic response at indicated concentrations of PsA (Figure 4). Furthermore, the expression level of caspases 3 and 7 were monitored in the cell lysate of PsA-treated and control cells. Caspases 3 and 7 are highly specific proteases involved in execution phase of cell apoptosis through proteolytic cleavage of target proteins involved in DNA fragmentation, chromatin condensation, and cytoskeletal damage. Our results revealed the significant upregulation of both caspases in a concentration-dependent manner (Figure 4), which emphasizes the apoptotic toxicity of PsA at indicated high doses. However, it is important to point out that the apoptotic effect of PsA on RWPE-1 prostatic cells is seen at approximately 10 and 20-fold its effective anticancer concentrations, signaling the broad safety profile of PsA at a cellular level [23].

The Up-and-Down procedure determined the LD_50_ for PsA to be greater than 550 mg/kg. This procedure was also utilized as a range finder to help determine which concentrations would be used in the 14-day single dose acute study. Complications with solubility prevented testing lethality of PsA at 2000 mg/kg. However, the therapeutic dose for prevention of tumor recurrence was determined to be 10 mg/kg body weight in a previous study [23], so the concentration chosen as the highest dose for this acute study was 50× higher, indicating a good safety. Both sexes of mice were implemented into this study as well as the 14-day acute toxicity study. This is in conjunction with the National Institutes of Health (NIH) guidelines (NOT-OD-16-006) that require the use of both sexes in screening of acute organ toxicity unless it is well justified. Gender-based differences in the toxicity of pharmaceuticals is a question that must be addressed to establish an accurate safety profile for all novel therapeutics.

The first noticeable indicators of possible toxicity in any mammalian safety assessment are changes in behavioral, autonomic, or neurological responses. Though slight hunching, reduced mobility, and anti-social behavior was noted within the first 1–2 h observation in a few of the male 500 mg/kg mice, only 3 h after dosing, the mice’s behavior returned to normal and remained healthy over the remainder of the 14-day experimental period. The assessment of animal welfare in safety studies often lacks experience and objectivity. Thus, there is the need for well-defined and implemented systems for recording animal indicators of distress [39,40,41]. To account for this, the mouse grimace scale was implemented to quantify pain and discomfort more reliably [28]. A senior toxicologist with over 20 years of animal testing supervised the initial 1–2 h observation after PsA dosing to monitor for changes in response.

A significant finding of the study was the extreme body weight loss of the female Swiss albino mice in the 500 mg/kg group over the 14-day safety study. Loss of body weight is widely accepted as a potential early marker for organ toxicity, and research shows a high empirical association between the loss of body weight and pathological findings [41]. Although many scientists define a body-weight reduction greater than 20% as a potential parameter for humane sacrifice, other recent studies strongly suggest a more flexible implementation of weight loss as a humane endpoint criteria that stresses using additional criteria for evaluating pain and puts more emphasis on the unique differences in experimental design [42]. In accordance with directive 2010/63/EU [43], which promotes the 3R’s (replacement, reduction, and refinement), the female mice 500 mg/kg group were closely monitored for the remainder of the study for behavior responses, neuromuscular responses, and autonomic responses. This group of female mice displayed no signs of pain and no changes in behavior from the time of initial dosing to the day of sacrifice. This guided the decision not to humanely sacrifice the mice after the weighing on day four showed >20% decrease in weight observed across all the animals in the group. Weight loss is an expected therapeutic outcome for PCSK9-targeting drugs. The phase IV clinical trial of the FDA-approved humanized mAb Repatha observed notable weight loss for people who have been taking the drug for 6–12 months, with the weight loss being especially prominent in females [44]. Another observation of note was that upon dissection, the PsA 250 mg/kg and 500 mg/kg treatment groups had no observable fat surrounding their G.I. tract, while the 10 mg/kg and control groups showed noticeable white fat surrounding their G.I. tract. This clearly indicates effective PsA PCSK9 axis-targeting outcomes at single high doses.

The AST and ALT are among the most reliable markers of liver cell injury/necrosis. The liver transaminases AST and ALT were both significantly increased in the female 250 mg/kg group compared to the control. The same increase was seen in the males but not to a significant degree. ALT is seen as the more specific marker for hepatotoxicity due to its high expression in hepatocytes and low expression in other cells of the body. In contrast, AST has mitochondrial and cytosolic forms present in the tissues of the kidneys, pancreas, lungs, brain, heart, skeletal muscle, and liver. AST and ALT levels can be elevated to levels exceeding 2000 U/L in cases of major hepatocyte injury or necrosis; however, increases not exceeding 5-fold the normal ranges are much more commonly seen in primary care medicine [45]. One study stated that serum ALT reference intervals for healthy Swiss albino mice are 46.15 ± 5.62 U/L for males and 59.03 ± 9.58 U/L for females [46]. Another study that measured the clinical markers for over 5000 healthy mice of different breeds found the average ALT for males to be 30 U/L and females to be 26 U/L, which are very close to the values observed in the control groups [47]. The average level of serum ALT for the 250 mg/kg female group was 49.5 ± 4.1. This was less than a two-fold increase over the serum ALT of the female control group (28.5 ± 3.6) and lower than the healthy reference level of 59.03 ± 9.58 U/L. Meanwhile, a cross-sectional study found an association between low LDL-C levels and elevated serum transaminases [48]. The histopathology of the liver tissue sections showed no signs of steatosis, necrosis, cirrhosis, inflammation, or other signs of toxicity to the hepatocytes. This leads to the belief that the increase in AST and ALT are not due to overt hepatocyte injury/necrosis.

Significantly lower levels of serum alkaline phosphatase (ALP) were observed in the female 250 mg/kg group. ALP is a hydrolase enzyme that is highly expressed in the liver, bone, and kidney. Elevated serum levels have commonly been used as a marker for hepatotoxicity. Lower ALP levels are not seen as a marker for hepatotoxicity but, instead, may indicate protein deficiency, malnutrition, or insufficient number of vitamins or minerals. The serum ALP levels of the female 250 mg/kg mice were not considered to be at very low levels (<30 U/L).

Liver is regarded as the organ with the highest levels of PCSK9 expression in the body [29], making it a primary target for testing PsA cytotoxicity on hepatocytes. The prosurvival Bcl-2 protein was slightly downregulated for the 500 mg/kg dose group in both animal genders, while it remained unchanged for the lower doses. On the other hand, the proapoptotic Bax remained unaffected at 10 and 250 mg/kg dose levels, while being upregulated at 500 mg/kg dose in both sexes (Figure 8). Taken together, the data strongly suggest that PsA could induce apoptosis in mouse liver tissues at very high doses relative to its previously reported therapeutically recommended dose of 10 mg/kg in nude mice [11,23]. In a similar fashion, the executioner caspases 3 and 7 were upregulated at the 500 mg/kg dose, while being unchanged for the 10 and 250 mg/kg doses of PsA (Figure 10). These results propose a broad biosafety margin for the PCSK9 axis-targeting PsA as a prostate cancer recurrence suppressor, with hepatocyte cell death observed only at 50-fold the recommended anticancer therapeutic dose [11,23].

Another sensitive indicator of potential drug toxicity is its effect on relative organ weight [49]. No significant changes (*p* > 0.05) on the relative liver, kidneys, spleen, lungs, heart, and brain weights were observed for the treatment and control groups of both mouse sexes. Similarly, no organ variations in the size, color, or overall appearance noticed in each treated group versus vehicle control.

Hematological parameters continue to be important markers in the detection of injury or disease in animals and humans. It has been reported that deleterious hematological results in animals have one of the highest concordances with negative results in humans [50]. The average RBC-C, HGB, HCT, MCV, and MCHC was unchanged amongst the treatment and control groups, indicating no signs of aplastic or hemolytic anemia. The platelet count increased in a step-wise manner in the 10 mg/kg and 250 mg/kg treatment groups, for both genders, versus vehicle control before falling slightly below the control levels in the 500 mg/kg treatment group. The PLT levels in the 500 mg/kg dosing group were too high to be considered thrombocytopenia [51]. The only significant finding among the hematological data was the increase in platelet count for the male 250 mg/kg group (*p* > 0.05). PsA treatment is causing thrombocytosis, an increase in platelet count, up to a certain dose; however, a high platelet count is not very indicative of serious injury and most times does not cause any symptoms. Possible causes of thrombocytosis are inflammation, anemia, or cancer [52].

The results of this 14-day acute toxicity study, when viewed together, strongly suggest that acute doses up to 500 mg/kg of PsA present no major toxicity to the organs analyzed. The decrease in body weight for the female 500 mg/kg group was the most significant finding and signaled potential organ toxicity. However, the relative organ weight saw no change compared to the control and the results of the histopathological examination revealed no signs of damage to the liver, kidney, heart, brain, and lungs. The weight loss effect of PsA is expected in PCSK9-targeting entities. The hematological and biochemical parameters tested also showed miniscule change compared to the control, with the exception of the liver transaminases in the female 250 mg/kg dosing group. Future repeated dose studies will test the sub-acute and sub-chronic effects associated with PsA dosing. Moreover, HPLC detection of PsA in Swiss albino mouse sera inevitably confirmed systemic effects (Figure S3). The novel marine natural product PsA is a prospective entity appropriate for subsequent preclinical development of prostate and breast cancer recurrence controls.

## 4. Materials and Methods

### 4.1. Cell Lines and Culture Conditions

Non-tumorigenic human prostatic cells RWPE-1 and colon CCD 841 CoN epithelial cells were purchased from the American Type Culture Collection (ATCC, Manassas, VA, USA). RWPE-1 cells were maintained in Gibco keratinocyte serum-free basal medium (Thermo Scientific, Rockford, IL, USA) supplemented with 5 µg/mL bovine pituitary extract (BPE), and 5 ng/mL recombinant human EGF. CCD 841 CoN cells were cultured in EMEM (ATCC, Manassas, VA, USA) supplemented with 10% fetal bovine serum (R&D Systems, Inc, Minneapolis, MN, USA). Cells were regularly sub-cultured upon confluency using trypsin EDTA (Corning, Glendale, AZ, USA) and fresh growth media. Cells were maintained in a humidified incubator kept at 37 °C with 5% CO_2_.

### 4.2. In Vitro Cytotoxicity of Pseurotin A

#### 4.2.1. MTT-Based Cell Cytotoxicity Assay

RWPE-1 cells were plated in a 96-well microplate at a density of 3 × 10^4^ cells per well. Cells were allowed to recover and attach for 24 h post seeding, while CCD 841 CoN were plated at a cell density of 2 × 10^4^ per well and allowed 12 h to adhere. Upon attaching, media were removed, and serum-free media containing different treatment concentration of PsA (from a stock solution of 100 mM in sterile DMSO) were added to each well. Cells were then incubated with treatment concentrations for 24 h, and then media were gently removed, and 100 µL of MTT solution (0.5 mg/mL final concentration) were added for each well. Plates were then incubated for 3 h and monitored for the formation of formazan crystals. After the incubation period, media were gently removed, and crystals were solubilized in 100 µL DMSO before measuring the optical absorbance at 570 nm using a Synergy 2 microplate reader (Bio Tek Instruments Inc., Winooski, VT, USA).

#### 4.2.2. ATP-Based Cell Cytotoxicity Assay

RWPE-1 cells were plated in a 96-well microplate at a density of 3 × 10^4^ cells per well. Cells were allowed to recover and attach for 24 h post seeding and 3 replicates/groups were utilized. Upon attaching, media were removed, and serum-free media containing different treatment concentration of PsA (from a stock solution of 500 mM in sterile DMSO) added to each well. Cells were then incubated with treatment concentrations for 24 h after which ATP release was determined using a Promega CellTiter-Glo^®^ 2.0 Assay kit (Promega Corporation, Madison, WI, USA). An amount of 95 μL of CellTiter-Glo^®^ 2.0 Reagent (equal to the volume of cell culture media resent in each well) was added to each well containing cells. After 2 min on an orbital shaker followed by another 10 min incubation at rt, the luminescence of each well was measured using a Synergy H1 hybrid reader (BioTek Instruments Inc., Winooski, VT, USA).

#### 4.2.3. Western Blot Analysis

Cells treated with different concentrations of PsA and liver tissues isolated from animals of different treatment and control groups were lysed using RIPA buffer (Thermo Scientific, Rockford, IL, USA) supplemented with a mammalian protease inhibitor cocktail (G-Biosciences, St. Louis, MO, USA). Protein concentrations in cell and tissue lysates were determined by Pierce BCA assay (Thermo Scientific, Rockford, IL, USA) as per manufacturer protocol. Lysates were mixed with equal volumes of 2X Laemmli buffer (Bio-Rad, Hercules, CA, USA) containing 5% β-mercaptoethanol before heat blocking in a water bath at 90 °C for 5 min. About 30 ug of cell and tissue lysates were resolved on 10 and 12% SDS-PAGE using Tris/Glycine/SDS as resolving buffer. Resolved protein bands were then transferred to PVDF membranes using Tris/Glycine buffer in 20% methanol/water at 4 °C overnight. Membranes were then blocked by rocking in 5% BSA/TBST for 2 h. Blocked membranes were then incubated overnight with different primary antibodies at 4 °C. Tubulin, Bax, Bcl-2 antibodies were purchased from Cell Signaling (Danvers, MA, USA) and caspases 3 and 7 were obtained from ProteinTech (Rosemont, IL, USA). Primary antibodies were used per vendor recommended dilutions in 5% BSA/TBST. Membranes were then washed 3–4 times with TBST before incubating with Cell Signaling HRP-linked antirabbit secondary antibody for an additional 2 h. Finally, membranes were washed 3–4 times with TBST before adding Super Signal West Femto prior to the imaging with ChemiDoc Touch Imaging System (Bio-Rad, Hercules, CA, USA). Densitometric analyses accomplished using the Image J software (NIH, Bethesda, MD, USA). Photographed protein blots were then normalized to β-tubulin for a quantitative comparison relative to untreated control cells and tissues.

### 4.3. Experimental Animals

Twenty male and twenty female Swiss albino mice, 4–5 weeks old, were purchased from Envigo (Indianapolis, IN, USA). The animals were given a 2-week acclimatization period and maintained under clean room conditions with a relative humidity of 55–65%, a temperature of 22 ± 2 °C, a 12:12 h light/dark cycle, Alpha-Dri bedding, and free access to drinking water and pelleted rodent chow (no. 7012, Envigo/Teklad, Madison, WI, USA). Animals were housed in group cages (male; n = 5 and female; n = 5) and kept in the same environmental conditions. All animal experiments were approved by the Institutional Animal Care and Use Committee (IACUC), University of Louisiana at Monroe, and were conducted in strict accordance with good animal practice as defined by NIH guidelines (Protocol # 21DEC-KES-03).

### 4.4. Up-and-Down Procedure

The procedure for determination of an LD_50_ was performed following the Organization of Economic Co-operation and Development (OECD) guideline 425 [27] for the testing of chemicals. Animals were given a 2-week acclimatization period. The starting dose for the procedure was 175 mg/kg per guideline 425 suggestion for the testing small molecules. The dosing progression was 175–550 mg/kg and was halted there due to limited PsA solubility at higher concentrations. Initially, mice were randomly selected and treated with a single PsA oral dose via oral gavage feeding syringe. After administering the dose, the mice were observed for mortality/morbidity and behavioral signs of pain/toxicity at 1, 2, 4, 6, 12, and 24 h and then once daily for a total of 14 days. If no mortality was observed after 24 h, another animal would receive a 3.2× increased dose, in a step wise manner, until stopping criteria was met (the upper limit of 550 mg/kg was reached). Toxicity was observed for 14 days unless clear signs of grimace and pain were present. In that case, the animal was euthanized in accordance with the 3R’s of toxicity testing. Rats were anesthetized using Isoflurane (2–5%).

### 4.5. 14-Day Single Oral Dose PsA Acute Toxicity Study Design

For the single oral dose 14-day acute toxicity study, healthy 8-week-old male and female Swiss albino mice were randomly selected and placed in 8 groups (n = 5/sex/group). Graded doses of PsA (10, 250, 500 mg/kg body weight) were administered to the mice formulated in <5% DMSO/0.2% Tween 80 at a volume not exceeding 200 µL by flexible plastic oral gavage (2 mm diameter with stainless steel bite protector, 18-gauge, 3.81 cm long). The mice were observed for mortality, morbidity, and behavioral signs of pain/toxicity at 1, 2, 4, 6, 12, and 24 h and then once daily for a total of 14 days. The body weights of the mice were weighed in grams using a Scout^TM^ Pro (Ohaus Corp., Pine Brook, NJ, USA) on day 0, 4, 8, 11, 14 after initial treatment. On day 14, all mice were anesthetized using isoflurane (USP-vaporizer method, Matrix VIP-3000, exposure of animals in anesthesia chamber at 3% isoflurane vaporization rate. Exposure time 3 min). Mice were then euthanized by cervical dislocation according to AVMA Guidelines on Euthanasia (2013) and dissected. Mice organs (brain, liver, kidney, heart, lungs, spleen) were excised and weighed for histopathological examination. The organs were stored in 10% formalin for 24 h then transferred to 70% ethanol. This study was performed following the OECD guideline 423 for the testing of chemicals [53].

### 4.6. Hematological and Biochemical Evaluation

Sacrificed mice were decapitated and the blood was quickly collected into a green topped Sodium Heparin 3.6 mg blood collection tube (BD Vacutainer REF# 368771) as well as a purple topped K2 EDTA (K2E) 3.6 mg blood collection tube (BD Vacutainer REF# 367841) to ensure that all blood samples were free of clots. The Sodium Heparin samples were immediately centrifuged at 13,000× *g* for 10 min and the plasma was collected for biochemical analysis. Samples were analyzed and glucose, AST, ALT, ALP, BUN, and creatinine levels were determined with the Beckman AU680 clinical chemistry analyzer system (Beckman Coulter, Atlanta, GA, USA). The EDTA samples were analyzed for hematological parameters and WBC, RBC, Hgb, Hct, MCV, MCHC, and PLT were determined using the Siemens Advia 120 hematology analyzer (Siemens Healthcare Diagnostics Inc., Tarrytown, NY, USA). All blood samples were analyzed at the LSU School of Veterinary Medicine (SVM) Clinical Pathology Laboratory at Baton Rouge, Louisiana.

### 4.7. Data Analysis

Results for the hematological and biochemical parameters were analyzed separately by a one-way Analysis of Variance (ANOVA) followed by multiple comparison with Dunnett’s test. All statistical analysis was performed using GraphPad Prism version 8 software (San Diego, CA, USA). Data in this study was expressed as a mean ± SD (standard deviation) and mean ± SEM (standard error of mean). A probability value of <0.05 was considered statistically significant (* *p* < 0.05; ** *p* < 0.01; *** *p* < 0.001; and **** *p* < 0.0001).

### 4.8. Paraffin Embedding and Staining

The mouse organs were carefully removed and quickly fixed in 10% neutral buffered formalin for 24 h before transfer to 70% ethanol. Organs were then embedded in paraffin before sectioning and staining by hematoxylin and eosin (H&E). All sectioning and staining were done at the AML Laboratories (Jacksonville, FL, USA). The paraffin-embedded tissues were sliced into 5 um-thick sections before being mounted on a positively charged slide. The 5 um-thick sections were then dewaxed using xylene, rinsed using 80–95% ethanol, and rehydrated using H_2_O. Afterwards they were stained with H&E before being dehydrated again using 95–80% ethanol to xylene.

## Figures and Tables

**Table 1:** Relative organ weight (mean ± SD) of PsA versus control-treated male Swiss albino mice. After being excised, intact organs were dabbed with a cloth to remove excess blood and immediately weighed. Kidneys and lungs were weighed together. Data shown as mean ± SD for n = 5/sex/group.

Parameters	Control	10 mg/kg	250 mg/kg	500 mg/kg
Liver	1819 ± 107.3	1968 ± 330.1	1705 ± 196.9	1664 ± 306.5
Kidneys	858.4 ± 57.3	730.8 ± 106.4	714.6 ± 44.4	756.0 ± 148.3
Spleen	133.4 ± 20.4	124.4 ± 7.4	117.2 ± 13.2	141.3 ± 32.4
Lungs	263.0 ± 40.0	350.2 ± 80.1	278.8 ± 57.8	341.5 ± 62.8
Heart	223.4 ± 9.2	285.8 ± 86.2	220.6 ± 18.4	295.3 ± 25.4
Brain	346.5 ± 68.9	405.2 ± 63.1	397.8 ± 72.2	458.0 ± 24.2

**Table 2:** Relative organ weight (mean ± SD) of PsA versus control-treated female Swiss albino mice. After being excised, intact organs were dabbed with a cloth to remove excess blood and immediately weighed. Kidneys and lungs were weighed together. Data shown as mean ± SD for n = 5/sex/group.

Parameters	Control	10 mg/kg	250 mg/kg	500 mg/kg
Liver	1150 ± 90.6	1157 ± 119.0	1227 ± 18.0	1145 ± 240.4
Kidneys	435.6 ± 47.9	408.5 ± 33.1	437.2 ± 28.4	391.8 ± 50.9
Spleen	107.0 ± 28.2	123.0 ± 11.4	118.4 ± 6.1	104.4 ± 22.6
Lungs	258.4 ± 79.8	233.3 ± 39.1	248.2 ± 73.4	209.0 ± 51.6
Heart	165.4 ± 24.5	171.0 ± 51.2	184.4 ± 29.6	180.4 ± 26.8
Brain	443.6 ± 50.9	392.0 ± 12.7	428.0 ± 72.7	378.8 ± 33.5

**Table 3:** Comparison of PsA versus vehicle control-treated hematological biochemical parameters glucose (GLU), aspartate aminotransferase (AST), alanine aminotransferase (ALT), alkaline phosphatase (ALP), blood urea nitrogen (BUN), and creatinine (CREAT) in male Swiss albino mice. Data shown as mean ± SEM for n = 5/sex/group.

Parameters	Control	10 mg/kg	250 mg/kg	500 mg/kg
GLU (mg/dL)	224.0 ± 4.8	294.0 ± 15.8	234.3 ± 11.8	236.8 ± 7.7
AST (U/L)	93.0 ± 10.2	115.0 ± 17.3	153.8 ± 8.8	105.8 ± 19.7
ALT (U/L)	31.8 ± 0.8	31.5 ± 2.9	41.8 ± 2.7	29.3 ± 1.2
ALP (U/L)	74.8 ± 1.4	70.8 ± 5.0	79.5 ± 4.1	76.8 ± 9.1
BUN (mg/dL)	24.3 ± 0.2	21.0 ± 1.6	22.8 ± 1.5	20.3 ± 0.5
CREAT (mg/dL)	0.2 ± 0.0	0.2 ± 0.0	<0.2 ± 0.0	0.2 ± 0.0

**Table 4:** Comparison of PsA versus vehicle control-treated hematological biochemical parameters glucose (GLU), aspartate aminotransferase (AST), alanine aminotransferase (ALT), alkaline phosphatase (ALP), blood urea nitrogen (BUN), and creatinine (CREAT) in female Swiss albino mice. Data shown as mean ± SEM for n = 5/sex/group.

Parameters	Control	10 mg/kg	250 mg/kg	500 mg/kg
GLU (mg/dL)	198.5 ± 17.2	222.5 ± 20.5	221.5 ± 6.3	191.7 ± 4.0
AST (U/L)	78.0 ± 28.1	90.5 ± 5.9	241.0 ± 35.0	158.3 ± 4.5
ALT (U/L)	28.5 ± 3.6	29.5 ± 1.5	49.5 ± 4.1	40.5 ± 2.5
ALP (U/L)	111.0 ± 8.0	95.0 ± 5.2	68.5 ± 4.7	87.0 ± 8.5
BUN (mg/dL)	17.5 ± 1.2	19.3 ± 1.4	23.0 ± 1.3	21.7 ± 0.5
CREAT (mg/dL)	<0.2 ± 0.0	0.2 ± 0.0	<0.2 ± 0.0	<0.2 ± 0.0

**Table 5:** Comparison of PsA versus vehicle control-treated hematological parameters white blood cell count (WBC), red blood cell count (RBC), hemoglobin count (HGB), hematocrit (HCT), mean corpuscular volume (MCV), mean corpuscular hemoglobin concentration (MCHC), and platelet count (PLT) in male Swiss albino mice. Data shown as mean ± SEM for n = 5/sex/group.

Parameters	Control	10 mg/kg	250 mg/kg	500 mg/kg
WBC (10^3^/μL)	5.3 ± 1.2	6.0 ± 0.8	6.3 ± 1.9	4.1 ± 1.6
RBC (10^6^/μL)	9.0 ± 0.7	8.9 ± 0.4	9.0 ± 0.9	8.6 ± 3.4
HGB (g/dL)	13.2 ± 0.5	13.2 ± 0.6	13.2 ± 0.7	12.8 ± 1.2
HCT (%)	44.3 ± 4.8	45.1 ± 3.1	44.7 ± 5.3	41.3 ± 4.6
MCV (fL)	49.1 ± 2.1	50.5 ± 2.7	49.6 ± 2.8	48.0 ± 2.6
MCHC (g/dL)	30.0 ± 2.3	29.3 ± 2.1	29.7 ± 2.3	30.9 ± 2.2
PLT (10^3^/μL)	537.8 ± 117.6	679.7 ± 139.7	768.5 ± 133.2	547.3 ± 159.9

**Table 6:** Comparison of PsA versus vehicle control-treated hematological parameters white blood cell count (WBC), red blood cell count (RBC), hemoglobin count (HGB), hematocrit (HCT), mean corpuscular volume (MCV), mean corpuscular hemoglobin concentration (MCHC), and platelet count (PLT) in female Swiss albino mice. Data shown as mean ± SEM for n = 5/sex/group.

Parameters	Control	10 mg/kg	250 mg/kg	500 mg/kg
WBC (10^3^/μL)	4.1 ± 1.2	4.6 ± 0.8	5.2 ± 1.9	3.1 ± 1.1
RBC (10^6^/μL)	9.1 ± 0.7	9.1 ± 0.4	9.5 ± 0.9	10.0 ± 0.3
HGB (g/dL)	13.5 ± 0.5	13.4 ± 0.6	14.1 ± 0.7	15.0 ± 0.5
HCT (%)	47.3 ± 4.8	45.0 ± 3.1	46.3 ± 5.3	50.7 ± 2.8
MCV (fL)	51.8 ± 2.1	49.5 ± 2.7	48.7 ± 2.8	50.6 ± 1.3
MCHC (g/dL)	28.7 ± 2.3	30.0 ± 2.1	30.5 ± 2.3	29.6 ± 0.9
PLT (10^3^/μL)	514.0 ± 117.6	587.5 ± 139.7	635.0 ± 133.2	409.0 ± 129.7

## Supplementary Materials

The following supporting information can be downloaded at: https://www.mdpi.com/article/10.3390/molecules28031460/s1, Figure S1: NMR spectra data for isolated pseurotin A. (a) ^1^H NMR spectrum of PsA in CDCl_3_ recorded at 400 MHz. (b) ^13^C NMR spectrum of PsA in CDCl_3_ recorded at 100 MHz. Figure S2: High-performance liquid chromatography (HPLC) chromatogram-aided purity assessment of isolated PsA A. Figure S3: High-performance liquid chromatography (HPLC) chromatogram evidencing the first detection of PsA in Swiss albino mice serum. Instrument: SHIMADZU; column: COSMOSIL C-18 (4.6 ID × 250 mm); mobile phase: 0.1% HCOOH in water: acetonitrile (60:40) isocratic; UV detection: λ = 254 nm.

## References

[1] Seidah N.G., Prat A. (2012). The biology and therapeutic targeting of the proprotein convertases. Nat. Rev. Drug Discov..

[2] Hochholzer W., Giugliano R.P. (2010). Lipid lowering goals: Back to nature?. Ther. Adv. Cardiovasc. Dis..

[3] LaRosa J.C., Pedersen T.R., Somaratne R., Wasserman S.M. (2013). Safety and effect of very low levels of low-density lipoprotein cholesterol on cardiovascular events. Am. J. Cardiol..

[4] Dietschy J.M., Turley S.D. (2004). Thematic review series: Brain Lipids. Cholesterol metabolism in the central nervous system during early development and in the mature animal. J. Lipid Res..

[5] Rousselet E., Marcinkiewicz J., Kriz J., Zhou A., Hatten M.E., Prat A., Seidah N.G. (2011). PCSK9 reduces the protein levels of the LDL receptor in mouse brain during development and after ischemic stroke. J. Lipid Res..

[6] Moşteoru S., Gaiţă D., Banach M. (2020). An update on PCSK9 inhibitors—Pharmacokinetics, drug interactions, and toxicity. Expert Opin. Drug Metab. Toxicol..

[7] Mora C., Tittensor D.P., Adl S., Simpson A.G., Worm B. (2011). How many species are there on Earth and in the ocean?. PLoS Biol..

[8] Copmans D., Rateb M., Tabudravu J.N., Pérez-Bonilla M., Dirkx N., Vallorani R., Diaz C., Pérez del Palacio J., Smith A.J., Ebel R. (2018). Zebrafish-Based Discovery of Antiseizure Compounds from the Red Sea: Pseurotin A2 and Azaspirofuran A. ACS Chem. Neurosci..

[9] Jaspars M., De Pascale D., Andersen J.H., Reyes F., Crawford A.D., Ianora A. (2016). The marine biodiscovery pipeline and ocean medicines of tomorrow. J. Mar. Biol. Assoc. U. K..

[10] Bloch P., Tamm C., Bollinger P., Petcher T.J., Weber H.P. (1976). Pseurotin, a new metabolite of *Pseudeurotium ovalis* Stolk having an unusual hetero-spirocyclic system. Helv. Chim. Acta.

[11] Abdelwahed K.S., Siddique A.B., Mohyeldin M.M., Qusa M.H., Goda A.A., Singh S.S., Ayoub N.M., King J.A., Jois S.D., El Sayed K.A. (2020). Pseurotin A as a novel suppressor of hormone dependent breast cancer progression and recurrence by inhibiting PCSK9 secretion and interaction with LDL receptor. Pharmacol. Res..

[12] Hayashi Y., Shoji M., Yamaguchi S., Mukaiyama T., Yamaguchi J., Kakeya H., Osada H. (2003). Asymmetric Total Synthesis of Pseurotin A. Org. Lett..

[13] Radišauskas R., Kuzmickienė I., Milinavičienė E., Everatt R. (2016). Hypertension, serum lipids and cancer risk: A review of epidemiological evidence. Medicina.

[14] Murai T. (2015). Cholesterol lowering: Role in cancer prevention and treatment. Biol. Chem..

[15] Emberson J.R., Kearney P.M., Blackwell L., Newman C., Reith C., Bhala N., Holland L., Peto R., Keech A., Collins R. (2012). Lack of effect of lowering LDL cholesterol on cancer: Meta-analysis of individual data from 175,000 people in 27 randomized trials of statin therapy. PloS ONE.

[16] Ravnskov U., McCully K.S., Rosch P.J. (2012). The statin-low cholesterol-cancer conundrum. QJM Mon. J. Assoc. Physicians.

[17] Pelton K., Freeman M.R., Solomon K.R. (2012). Cholesterol and prostate cancer. Curr. Opin. Pharmacol..

[18] Zhuang L., Kim J., Adam R.M., Solomon K.R., Freeman M.R. (2005). Cholesterol targeting alters lipid raft composition and cell survival in prostate cancer cells and xenografts. J. Clin. Investig..

[19] Wang X., Sun B., Wei L., Jian X., Shan K., He Q., Huang F., Ge X., Gao X., Feng N. (2022). Cholesterol and saturated fatty acids synergistically promote the malignant progression of prostate cancer. Neoplasia.

[20] Ghanbari F., Mader S., Philip A. (2020). Cholesterol as an endogenous ligand of ERRα promotes ERRα-mediated cellular proliferation and metabolic target gene expression in breast cancer cells. Cells.

[21] Garcia-Estevez L., Moreno-Bueno G. (2019). Updating the role of obesity and cholesterol in breast cancer. Breast Cancer Res..

[22] Li C., Yang L., Zhang D., Jiang W. (2016). Systematic review and meta-analysis suggest that dietary cholesterol intake increases risk of breast cancer. Nutr. Res..

[23] Abdelwahed K.S., Siddique A.B., Qusa M.H., King J.A., Souid S., Abd Elmageed Z.Y., El Sayed K.A. (2021). PCSK9 Axis-targeting pseurotin A as a novel prostate cancer recurrence suppressor lead. ACS Pharmacol. Transl. Sci..

[24] Robinson J.G., Farnier M., Krempf M., Bergeron J., Luc G., Averna M., Stroes E.S., Langslet G., Raal F.J., El Shahawy M. (2015). Efficacy and safety of alirocumab in reducing lipids and cardiovascular events. N. Engl. J. Med..

[25] Tabrizi M., Bornstein G.G., Suria H. (2010). Biodistribution mechanisms of therapeutic monoclonal antibodies in health and disease. AAPS J..

[26] Koren M.J., Sabatine M.S., Giugliano R.P., Langslet G., Wiviott S.D., Ruzza A., Ma Y., Hamer A.W., Wasserman S.M., Raal F.J. (2019). Long-Term Efficacy and Safety of Evolocumab in Patients with Hypercholesterolemia. J. Am. Coll. Cardiol..

[27] OECD (2022). Test No. 425: Acute Oral Toxicity: Up-and-Down Procedure.

[28] Langford D.J., Bailey A.L., Chanda M.L., Clarke S.E., Drummond T.E., Echols S., Glick S., Ingrao J., Klassen-Ross T., Lacroix-Fralish M.L. (2010). Coding of facial expressions of pain in the laboratory mouse. Nat. Methods.

[29] Zaid A., Roubtsova A., Essalmani R., Marcinkiewicz J., Chamberland A., Hamelin J., Tremblay M., Jacques H., Jin W., Davignon J. (2008). Proprotein convertase subtilisin/kexin type 9 (PCSK9): Hepatocyte-specific low-density lipoprotein receptor degradation and critical role in mouse liver regeneration. Hepatology.

[30] Da Dalt L., Castiglioni L., Baragetti A., Audano M., Svecla M., Bonacina F., Pedretti S., Uboldi P., Benzoni P., Giannetti F. (2021). PCSK9 deficiency rewires heart metabolism and drives heart failure with preserved ejection fraction. Eur. Heart J..

[31] National Toxicology Program (2019). The Digitized Atlas of Mouse Liver Lesions.

[32] Gartner L., Hiatt J., Coryell P. (1994). Color Atlas of Histology.

[33] Hesketh E.E., Czopek A., Clay M., Borthwick G., Ferenbach D., Kluth D., Hughes J. (2014). Renal ischaemia reperfusion injury: A mouse model of injury and regeneration. J. Vis. Exp..

[34] Sachdeva J., Dai W., Kloner R.A. (2014). Functional and histological assessment of an experimental model of Takotsubo’s cardiomyopathy. J. Am. Heart Assoc..

[35] Jordan W.H., Young J.K., Hyten M.J., Hall D.G. (2011). Preparation and analysis of the central nervous system. Toxicol. Pathol..

[36] Taylor B.A., Thompson P.D. (2018). Statin-associated muscle disease: Advances in diagnosis and management. Neurotherapeutics.

[37] Tabernilla A., Dos Santos Rodrigues B., Pieters A., Caufriez A., Leroy K., Van Campenhout R., Cooreman A., Gomes A.R., Arnesdotter E., Gijbels E. (2021). In vitro liver toxicity testing of chemicals: A Pragmatic approach. Int. J. Mol. Sci..

[38] Lomakina G.Y., Modestova Y.A., Ugarova N.N. (2015). Bioluminescence assay for cell viability. Biochemistry/Biokhimiia.

[39] Oliver V.L., Thurston S.E., Lofgren J.L. (2018). Using Cageside measures to evaluate analgesic efficacy in mice (*Mus musculus*) after Surgery. J. Am. Assoc. Lab. Anim. Sci..

[40] Hawkins P., Morton D.B., Burman O., Dennison N., Honess P., Jennings M., Lane S., Middleton V., Roughan J.V., Wells S. (2011). A guide to defining and implementing protocols for the welfare assessment of laboratory animals: Eleventh report of the BVAAWF/FRAME/RSPCA/UFAW Joint Working Group on Refinement. Lab. Anim..

[41] Silva A.V., Norinder U., Liiv E., Platzack B., Öberg M., Törnqvist E. (2021). Associations between clinical signs and pathological findings in toxicity testing. Altex.

[42] Talbot S.R., Biernot S., Bleich A., van Dijk R.M., Ernst L., Häger C., Helgers S.O.A., Koegel B., Koska I., Kuhla A. (2020). Defining body-weight reduction as a humane endpoint: A critical appraisal. Lab. Anim..

[43] The European Parliament, The Council of The European Union (2010). Directive 2010/63/Eu of The European Parliament and of the Council of 22 September 2010 on the Protection of Animals Used for Scientific Purposes. Off. J. Eur. Union.

[44] Amgen Double-blind, Randomized, Multicenter, Placebo-Controlled Study to Characterize the Efficacy, Safety, and Tolerability of 24 Weeks of Evolocumab for LDL-C Reduction in Pediatric Subjects 10 to 17 Years of Age with HeFH. https://clinicaltrials.gov/ct2/results?cond=Double-blind%2C+Randomized%2C+Multicenter%2C+Placebo-Controlled+Study+to+Characterize+the+Efficacy%2C+Safety%2C+and+Tolerability+of+24+Weeks+of+Evolocumab+for+LDL-C+Reduction+in+Pediatric+Subjects+10+to+17+Years+of+Age+with+HeFH&term=&cntry=&state=&city=&dist=.

[45] Giboney P.T. (2005). Mildly elevated liver transaminase levels in the asymptomatic patient. Am. Fam. Physician.

[46] Silva-Santana G., Bax J.C., Fernandes D.C.S., Bacellar D.T.L., Hooper C., Dias A., Silva C.B., de Souza A.M., Ramos S., Santos R.A. (2020). Clinical hematological and biochemical parameters in Swiss, BALB/c, C57BL/6 and B6D2F1 *Mus musculus*. Anim. Model. Exp. Med..

[47] Otto G.P., Rathkolb B., Oestereicher M.A., Lengger C.J., Moerth C., Micklich K., Fuchs H., Gailus-Durner V., Wolf E., Hrabě de Angelis M. (2016). Clinical Chemistry Reference Intervals for C57BL/6J, C57BL/6N, and C3HeB/FeJ Mice (*Mus musculus*). J. Am. Assoc. Lab. Anim. Sci..

[48] Jiang Z.G., Mukamal K., Tapper E., Robson S.C., Tsugawa Y. (2014). Low LDL-C and high HDL-C levels are associated with elevated serum transaminases amongst adults in the United States: A cross-sectional study. PLoS ONE.

[49] Zainal Z., Ong A., Yuen May C., Chang S.K., Abdul Rahim A., Khaza’ai H. (2020). Acute and subchronic oral toxicity of oil palm puree in Sprague-Dawley rats. Int. J. Environ. Res. Public Health.

[50] Olson H., Betton G., Robinson D., Thomas K., Monro A., Kolaja G., Lilly P., Sanders J., Sipes G., Bracken W. (2000). Concordance of the toxicity of pharmaceuticals in humans and in animals. Regul. Toxicol. Pharmacol..

[51] Morowski M., Vögtle T., Kraft P., Kleinschnitz C., Stoll G., Nieswandt B. (2013). Only severe thrombocytopenia results in bleeding and defective thrombus formation in mice. Blood.

[52] Scott J. (2022). When to Worry About High Platelet Count.

[53] OECD (2002). Test No. 423: Acute Oral toxicity—Acute Toxic Class Method.

