# Supplementary material for: Towards Developing Novel Prostate Cancer Recurrence Suppressors: Acute Toxicity of Pseurotin A, an Orally Active PCSK9 Axis-Targeting Small-Molecule in Swiss Albino Mice
Source: Molecules. 2023 Feb 2;28(3):1460. doi: 10.3390/molecules28031460 (PMC9922019; doi:10.3390/molecules28031460)
Supplement: Supplementary file 1 [file molecules-28-01460-s001.zip › molecules-2180548-supplementary.pdf]

Towards Developing Novel Prostate Cancer Recurrence Suppressors: Acute Toxicity of Pseurotin A, an Orally Active PCSK9 Axis-Targeting Small-Molecule in Swiss albino Mice

Oliver C. McGehee [1,2] Hassan Y. Ebrahim [1,2] Ashkan H. Rad [1], Khaldoun S. Abdelwahed [1], Ethar A. Mudhish [1], Judy A. King [3], Iman E. Helal [1,4], Sharon A. Meyer [1] and Khalid A. El Sayed [1,*]

[1] School of Basic Pharmaceutical and Toxicological Sciences, College of Pharmacy, University of Louisiana at Monroe, 1800 Bienville Drive, Monroe, LA 71201, USA; mcgeheoc@warhawks.ulm.edu (O.M.); ebrahim@ulm.edu (H.E.); hassankhanirada@warhawks.ulm.edu (A.R.); abdelwks@warhawks.ulm.edu (K.A.); mudhishea@warhawks.ulm.edu (E.M.); dr_eman_ezzat@mans.edu.eg (I.H.); meyer@ulm.edu (S.M.)

[2] Both coauthors contributed equally.

[3] Department of Integrated Medical Education, Thomas F. Frist, Jr. College of Medicine, Belmont University, 1900 Belmont Boulevard, Nashville, TN 37212, USA; judy.king@belmont.edu

[4] Current address: Department of Pharmacognosy, Faculty of Pharmacy, Mansoura University,
Mansoura 35516, Egypt

* Correspondence: elsayed@ulm.edu

Supplementary Material

Figure S1. NMR spectral data for isolated pseurotin A. (a) ^{1}H NMR spectrum of pseurotin A in $CDCl_3$ recorded at 400 MHz. (b) ^{13}C NMR spectrum of pseurotin A in $CDCl_3$ recorded at 100 MHz.

Figure S2. High-performance liquid chromatography (HPLC) chromatogram-aided purity assessment of isolated pseurotin A. Instrument: SHIMATZU; column: COSMOSIL C-18 (4.6 ID × 250 mm); mobile phase: 0.1 % HCOOH in water: acetonitrile (60:40) isocratic; UV detection: λ=254 nm.

Figure S3. High-performance liquid chromatography (HPLC) chromatogram evidencing the first detection of pseurotin A in Swiss albino mice plasma.

(a)

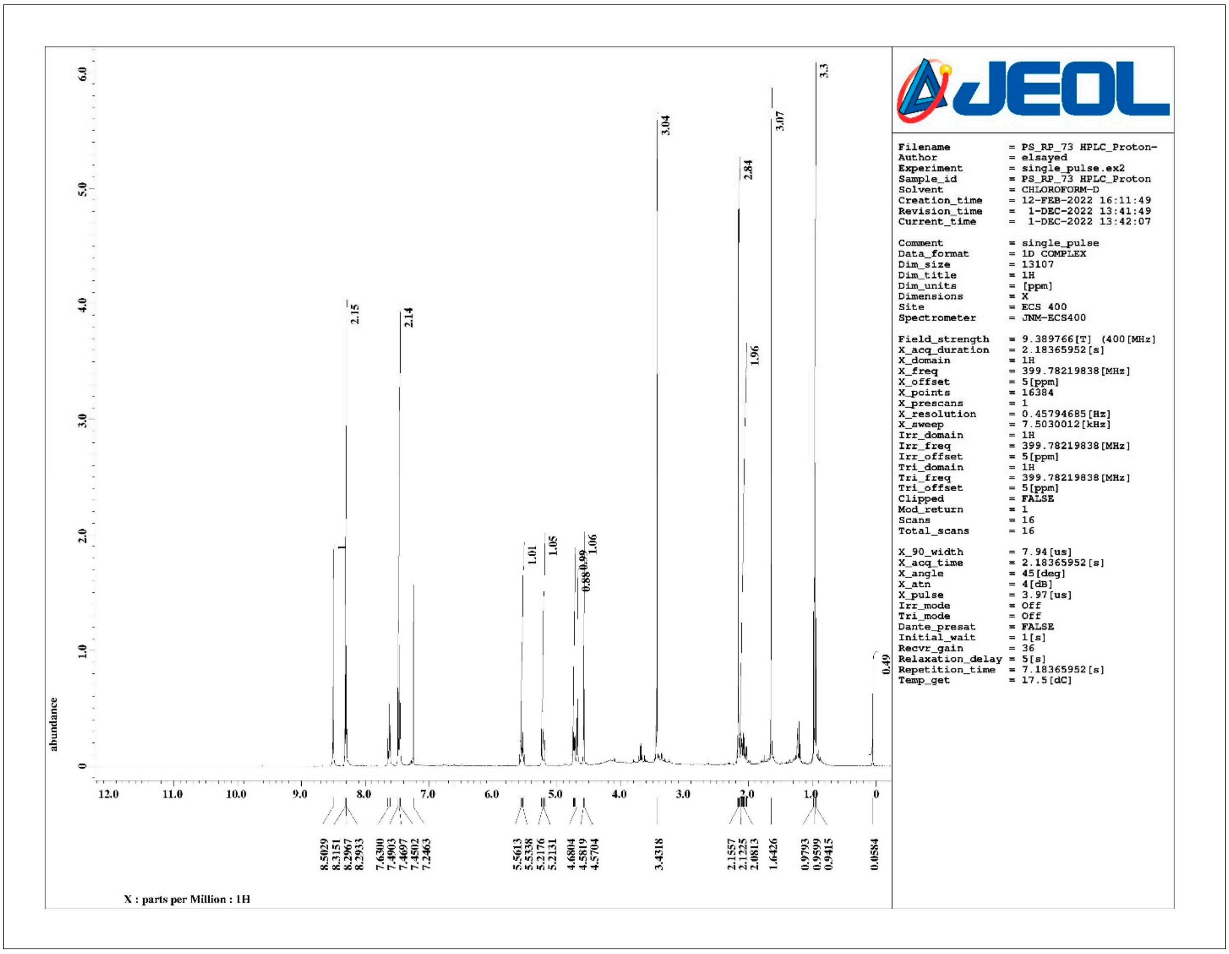
JEOL
Filename = PS_RP_73 HPLC_Proton-
Author = elsayed
Experiment = single_pulse.ex2
Sample_id = PS_RP_73 HPLC_Proton
Solvent = CHLOROFORM-D
Creation_time = 12-FEB-2022 16:11:49
Revision_time = 1-DEC-2022 13:41:49
Current_time = 1-DEC-2022 13:42:07
Comment = single_pulse
Data_format = 1D COMPLEX
Dim_size = 13107
Dim_title = 1H
Dim_units = [ppm]
Dimensions = X
Site = ECS 400
Spectrometer = JNM-ECS400
Field_strength = 9.389766[T] (400[MHz]
X_acq_duration = 2.18365952[s]
X_domain = 1H
X_freq = 399.78219838[MHz]
X_offset = 5[ppm]
X_points = 16384
X_prescans = 1
X_resolution = 0.45794685[Hz]
X_sweep = 7.5030012[kHz]
Irr_domain = 1H
Irr_freq = 399.78219838[MHz]
Irr_offset = 5[ppm]
Tri_domain = 1H
Tri_freq = 399.78219838[MHz]
Tri_offset = 5[ppm]
Clipped = FALSE
Mod_return = 1
Scans = 16
Total_scans = 16
X_90_width = 7.94[us]
X_acq_time = 2.18365952[s]
X_angle = 45[deg]
X_atn = 4[dB]
X_pulse = 3.97[us]
Irr_mode = Off
Tri_mode = Off
Dante_presat = FALSE
Initial_wait = 1[s]
Recvr_gain = 36
Relaxation_delay = 5[s]
Repetition_time = 7.18365952[s]
Temp_get = 17.5[dC]
abundance
X : parts per Million : 1H
2.15
2.14
1.01
1.05
0.99
0.88
1.06
3.04
2.84
1.96
3.07
3.3
0.49
8.5029
8.3151
8.2967
8.2933
7.6300
7.4903
7.4697
7.4502
7.2463
5.5613
5.5338
5.2176
5.2131
4.6804
4.5819
4.5704
3.4318
2.1557
2.1225
2.0813
1.6426
0.9793
0.9599
0.9415
0.0584

(b)

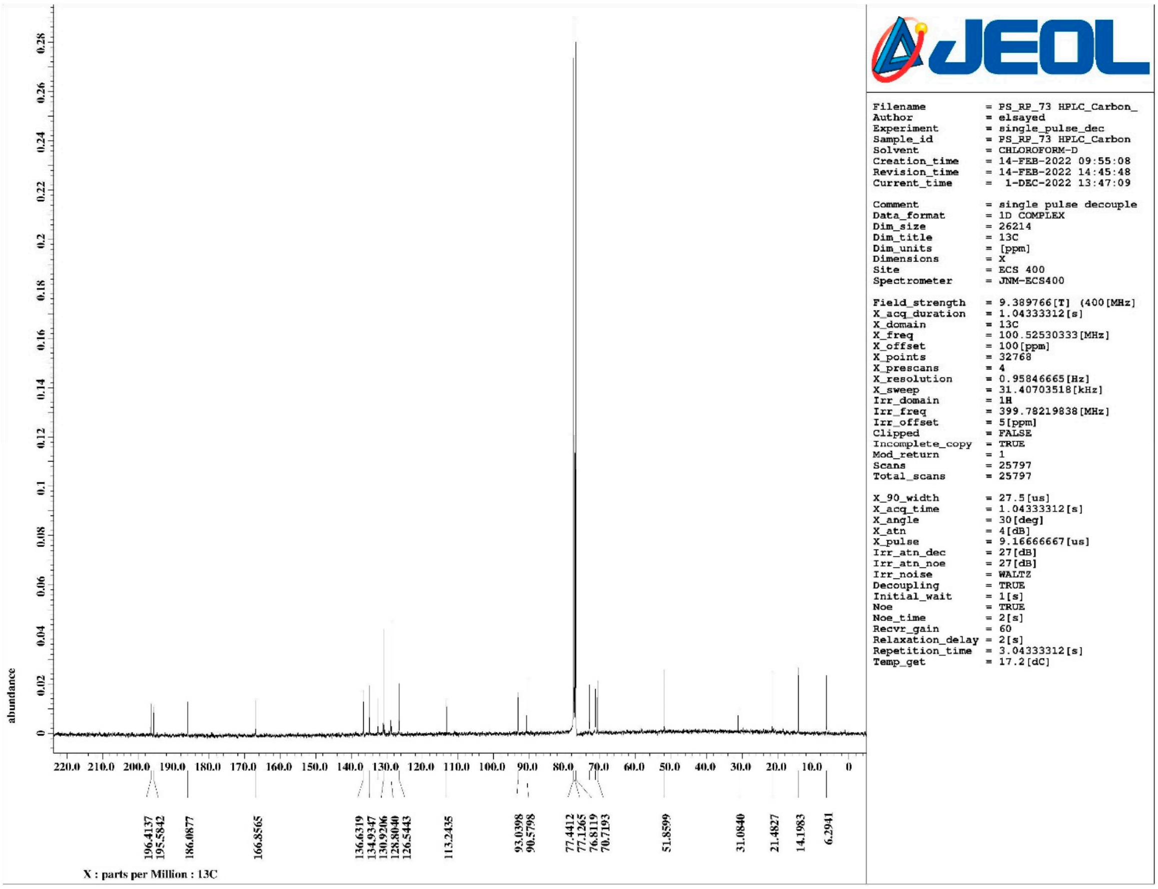

Figure S1. NMR spectra data for isolated pseurotin A. (**a**) ^{1}H NMR spectrum of pseurotin A in $CDCl_3$ recorded at 400 MHz. (**b**) ^{13}C NMR spectrum of pseurotin A in $CDCl_3$ recorded at 100 MHz.

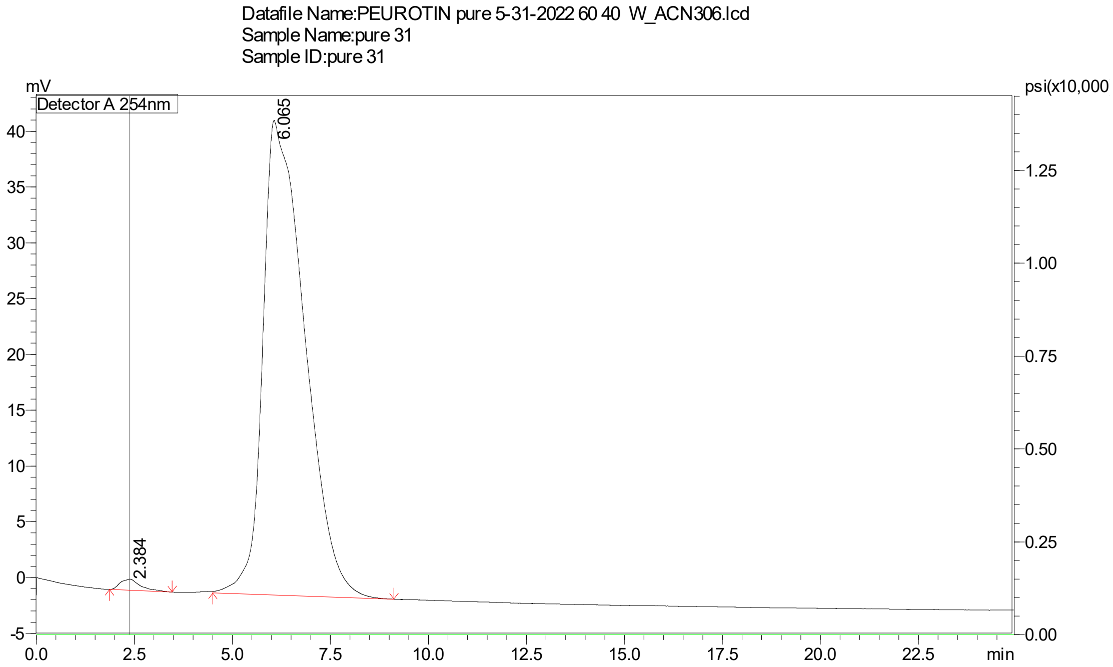

Figure S2. High-performance liquid chromatography (HPLC) chromatogram-aided purity assessment of isolated pseurotin A. Instrument: SHIMADZU; column: COSMOSIL C-18 (4.6 ID × 250 mm); mobile phase: 0.1 % HCOOH in water: acetonitrile (60:40) isocratic; UV detection: λ=254 nm.

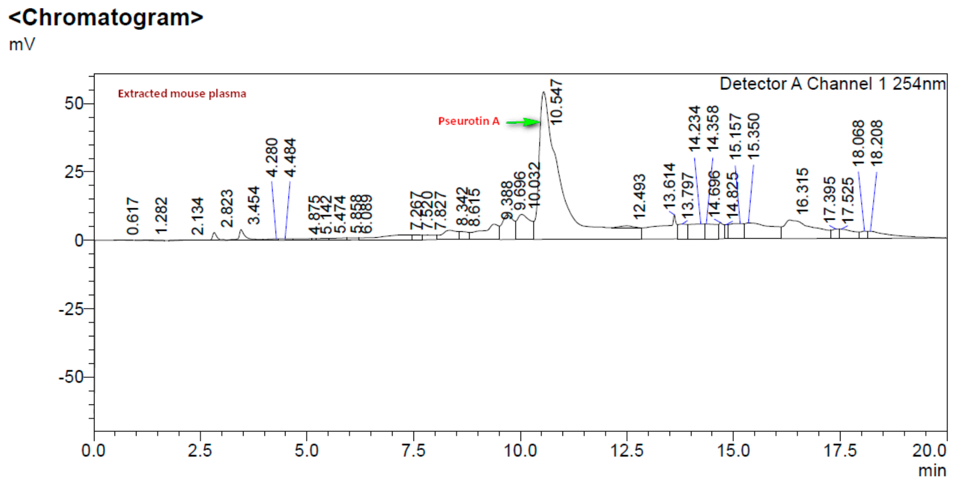

H_2O (0.1 % HCCOH)/ACN gradient elution

Figure S3. High-performance liquid chromatography (HPLC) chromatogram evidencing the first detection of pseurotin A in Swiss albino mice serum. Instrument: SHIMADZU; column: COSMOSIL C-18 (4.6 ID × 250 mm); mobile phase: 0.1 % HCOOH in water: acetonitrile (60:40) isocratic; UV detection: λ=254 nm.
